# Inefficient differentiation response to cell cycle stress leads to genomic instability and malignant progression of squamous carcinoma cells

**DOI:** 10.1038/cddis.2017.259

**Published:** 2017-06-29

**Authors:** Pilar Alonso-Lecue, Isabel de Pedro, Vincent Coulon, Rut Molinuevo, Corina Lorz, Carmen Segrelles, Laura Ceballos, Daniel López-Aventín, Ana García-Valtuille, José M Bernal, Francisco Mazorra, Ramón M Pujol, Jesús Paramio, J Ramón Sanz, Ana Freije, Agustí Toll, Alberto Gandarillas

**Affiliations:** 1 Cell Cycle, Stem Cell Fate and Cancer Laboratory, Instituto de Investigación Marqués de Valdecilla (IDIVAL), Santander, Spain; 2 Institut de Genétique Moléculaire de Montpellier, CNRS/UM2, Montpellier, France; 3 Molecular Oncology Unit, Department of Basic Research, Centro de Investigaciones Energéticas, Medioambientales y Tecnológicas (CIEMAT), CIBERONC, Madrid, Spain; 4 Department of Dermatology, Hospital del Mar, Barcelona, Spain; 5 Clínica Mompía, Mompía, Spain; 6 Department of Cardiovascular Surgery, Hospital Universitario Marqués de Valdecilla, Santander, Spain; 7 Department of Pathology, Hospital Universitario Marqués de Valdecilla, Santander, Spain; 8 Institut Hospital del Mar d'Investigacions Mèdiques, Barcelona, Spain; 9 Department of Plastic Surgery, Hospital Universitario Marqués de Valdecilla, Santander, Spain; 10 INSERM, Languedoc-Roussillon, Montpellier, France

## Abstract

Squamous cell carcinoma (SCC) or epidermoid cancer is a frequent and aggressive malignancy. However in apparent paradox it retains the squamous differentiation phenotype except for very dysplastic lesions. We have shown that cell cycle stress in normal epidermal keratinocytes triggers a squamous differentiation response involving irreversible mitosis block and polyploidisation. Here we show that cutaneous SCC cells conserve a partial squamous DNA damage-induced differentiation response that allows them to overcome the cell division block. The capacity to divide in spite of drug-induced mitotic stress and DNA damage made well-differentiated SCC cells more genomically instable and more malignant *in vivo*. Consistently, in a series of human biopsies, non-metastatic SCCs displayed a higher degree of chromosomal alterations and higher expression of the S phase regulator Cyclin E and the DNA damage signal *γ*H2AX than the less aggressive, non-squamous, basal cell carcinomas. However, metastatic SCCs lost the *γ*H2AX signal and Cyclin E, or accumulated cytoplasmic Cyclin E. Conversely, inhibition of endogenous Cyclin E in well-differentiated SCC cells interfered with the squamous phenotype. The results suggest a dual role of cell cycle stress-induced differentiation in squamous cancer: the resulting mitotic blocks would impose, when irreversible, a proliferative barrier, when reversible, a source of genomic instability, thus contributing to malignancy.

Squamous cell carcinoma (SCC) or epidermoid carcinoma arises in stratified epithelia including skin, head and neck, oesophagus, or cervix but also in simple epithelia (25–30% of lung cancer; http://www.cancer.org http://www.cancer.gov). SCCs are often aggressive and have poor prognosis causing significant death numbers. Although clinics and treatment of SCCs vary, they are mostly caused by external mutagenic agents (UV light, smoking, alcohol). Cutaneous SCC is not frequently fatal (4% metastasize)^1^ but is a paradigmatic and accessible model for the study of squamous cancer. The two types of cutaneous carcinoma: basal cell carcinoma (BCC; 80%) and SCC (20%) are the most common forms of cancer.^1, 2, 3, 4^ The comparison of BCC and SCC provides divergent lesions displaying different molecular alterations and paradoxical contrasting prognosis.^5^ While SCCs retain a squamous differentiated phenotype except for very dysplastic lesions,^6^ BCCs typically contain undifferentiated small cells reminiscent of proliferative basal keratinocytes although they display slow growth^7^ and a very low metastatic rate (0.1%).^8^ Why SCCs are more aggressive than skin BCCs, is a relevant clinical and biological question that remains unresolved.

We have previously revealed a novel epidermal response that triggers squamous differentiation upon cell cycle deregulation and DNA damage.^9, 10, 11, 12^ This DNA damage response (DDR) suppresses cell division but allows extra rounds of DNA replication resulting in polyploidy. This involves mitotic bypass (without intervening mitosis), mitotic slippage (defined as failure to arrest in G2/M)^13^ or acytokinetic mitosis.^12^ Due to this process, cell cycle stress induced in human keratinocytes by proto-oncogene MYC, the DNA replication regulator Cyclin E, or by inactivation of tumour suppressor p53, does not result in cellular transformation, but in a G2/M block that in turn induces squamous differentiation and polyploidy.^9, 10, 14^ We have proposed that this response constitutes an anti-oncogenic mechanism by which pre-cancerous cells are eliminated from the skin via squamous differentiation.^10, 11^ Consistently, in a large body of works on mouse skin differentiation prevailed upon overexpression of cell cycle or oncogenic molecules (for example, refs 15, 16, 17 reviewed in ref. 11).

Replication stress by causing DNA damage alerts cell cycle checkpoints of G2 and mitosis.^18, 19^ In keratinocytes, a mere impairment of the G2/M transitions induces massive terminal squamous differentiation and polyploidy and loss of proliferative potential within 48 h.^9, 14, 20^ This includes the inhibition of mitotic kinases (Aurora Kinase B, Polo-like Kinase, cdk1) or microtubuli (Nocodazole, Taxol) and also S phase defects (Doxorubicin or Bleomycine). We hypothesised that the capacity of SCC cells to divide upon replication stress in spite of polyploidy might contribute to malignancy. This would require alterations in mitosis control.^10^ Supporting this notion, overexpression of the global mitotic regulator FOXM1 drives the keratinocyte response to ectopic MYC or loss of p53 from increased differentiation to increased proliferation.^21^

We have now investigated the alterations of SCC and BCC cells in their response to cell cycle stress. We find that SCC but not BCC cells retain a partial squamous differentiation response to replication stress induced by Cyclin E. Analyses of a panel of human biopsies of BCC, non-metastatic SCC and metastatic SCC reveal significant differences in the expression of Cyclin E and in levels of DNA damage. In addition, we report that mitotic stress induced by consecutive mitosis blocks drive SCC cells to accumulate DNA damage, to progressively lose the squamous phenotype, to gain mesenchymal features and to become more malignant *in vivo*. We propose a model by which alterations in the squamous differentiation DDR at mitosis contributes to genomic instability and malignant progression of squamous cancer. This might contribute explaining why SCC tends to be aggressive in opposition to the less differentiated BCC.

## Results

**Figure 1:**
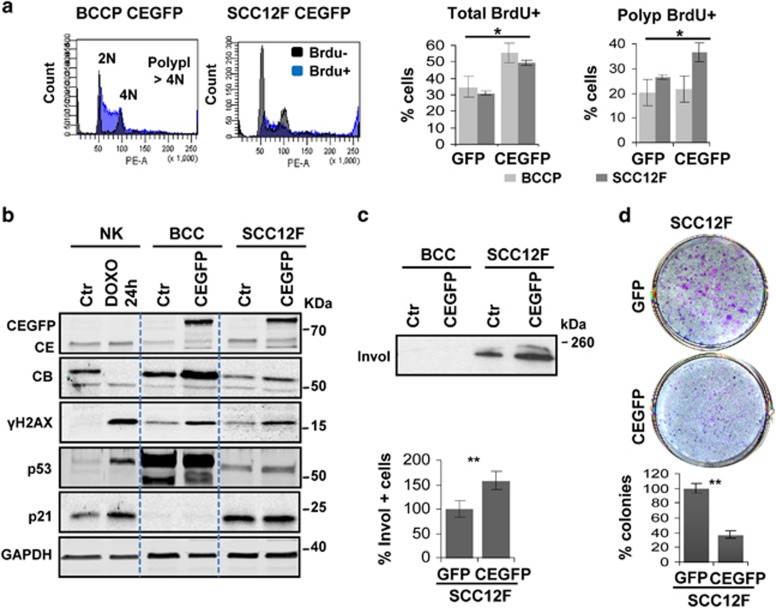
Cyclin E induces a partial squamous differentiation response in SCC12F cells and proliferation in BCCP cells. (**a**) Plots: representative cell cycle profiles (propidium iodide) of BCCP or SCC12F overexpressing Cyclin E-GFP (CEGFP) after a 1.5 h pulse of BrdU. BrdU negative (−) cells in black and BrdU positive cells (BrdU+) in blue. Bars: quantitation of total BrdU positive (+) cells or polyploid (>4N) BrdU+ cells in BCCP (light grey) or SCC12F (dark grey) overexpressing GFP or CEGFP. See also Supplementary Figure 1b. (**b**) Detection by western blot of CEGFP, Cyclin E (CE), Cyclin B (CB), *γ*H2AX, p53, or p21 in normal keratinocytes (NK) untreated (Ctr) or treated 24 h with doxorubicin (DOXO); or in BCC and SSC12F cells Ctr or overexpressing CEGFP. GAPDH is loading control. (**c**) Top: detection by western blot of involucrin in insoluble protein extracts (Invol); same number of cells per lane. Uncropped blots are shown in Supplementary Figure 12. Bottom: percent of SCC12F-CEGFP positive cells for Invol relative to SCC12F-GFP (as determined by immunofluorescence; Supplementary Figure 1d). (**d**) Top: clonogenic capacity of SCC12F-GFP and SCC12F-CEGFP, first passage after infection (1000 cells per well, wells are representative of triplicate samples; blue is the fibroblast feeder layer, pink the carcinoma cells); bottom: percent of proliferative colonies of SCC12F-CEGFP relative to SCC12F-GFP. Error bars are s.e.m. of duplicate or triplicate samples of at least two independent representative experiments. *P<0.05 and **P<0.01

Cyclin E accumulates during squamous differentiation and when overexpressed in proliferative keratinocytes it promotes replication stress, DNA damage, mitotic defects and polyploidy.^9, 20, 22, 23^ To study this response in carcinoma cells we overexpressed a GFP form of Cyclin E in cells originated from a human facial BCC (BCCP;^24^ Supplementary Figure 1a) or from a human facial well-differentiated SCC (SCC12F;^25^ Supplementary Figure 1a). Cyclin E induced DNA replication both in BCCP and SCC12F as monitored by BrdU incorporation (Figure 1a; Supplementary Figure 1b). However, whereas in BCCP this resulted in a boost of S phase, in SCC12F it promoted polyploidy. Cyclin E induced mitotic Cyclin B in BCCP, but not in SCC12F due to mitotic slippage or bypass (Figure 1b).^9^ As expected, Cyclin E also caused increase of the DNA damage marker *γ*H2AX both in BCCP and SCC12F (Figure 1b) due to replication stress.^9, 22^ BCCP display the mutated p53 antigen Pab240 (p53mut), whereas SCC12F are reported not to bear p53 mutations and did not express p53mut (Supplementary Figure 1c).^26^ In line with inactivating mutations which render the protein resistant to degradation,^27^ p53 was overexpressed in BCCP cells (Figure 1b; Supplementary Figure 1c). p53 transcriptional target p21CIP (p21) was barely detectable in proliferating BCCP *in vitro* (Figure 1b). p21 inhibits cdk2 and cell cycle entry or cdk1 and mitosis progression,^28^ for instance in response to DNA damage. In keratinocytes, p21 is transiently induced and binds cdk1 in the onset of squamous differentiation.^9, 29, 30^ Overexpression of Cyclin E in SCC12F cells caused a slight induction of p53 typical of DNA damage (Figure 1b).^9^ However, p21 was high both in parental SCC12F cells and upon ectopic Cyclin E, as compared with normal keratinocytes (Figure 1b). p21 can be expressed independently of p53 and its deregulation in SCC12F might reflect cell cycle alterations.

Overexpression of Cyclin E induced at some extent squamous differentiation in SCC12F, but not in BCCP, as measured by the squamous marker involucrin (Figure 1c; Supplementary Figure 1d). As we found no signs of apoptosis (Figure 1a; Supplementary Figure 1b), the induction of terminal differentiation is consistent with the significant loss of clonogenic potential of SCC12F-Cyclin E (Figure 1d). Growing SCC12F cells overexpressing Cyclin E after three passages continued to show higher DNA damage (Supplementary Figure 1e) and reduced clonogenic capacity than parental cells (Supplementary Figure 1f).

**Figure 2:**
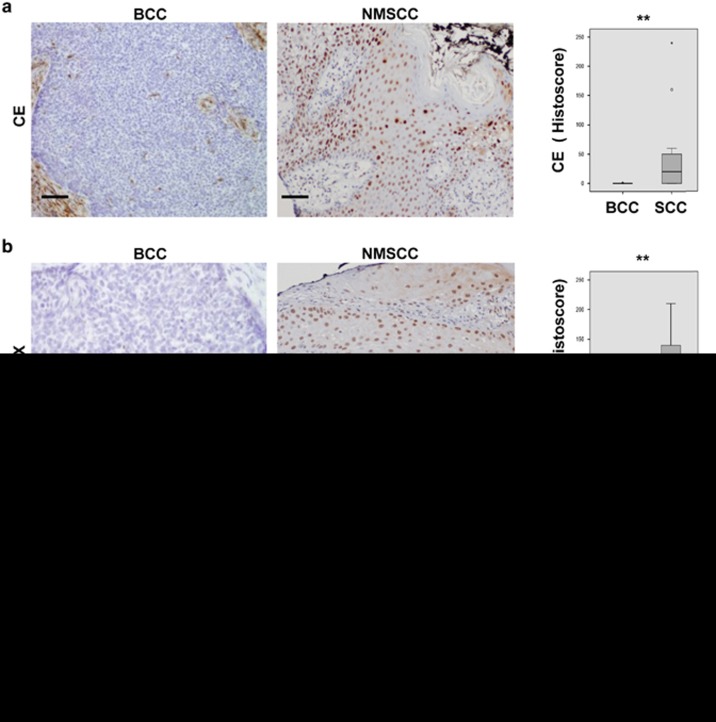
The axis Cyclin E/*γ*H2AX is high in human NMSCCs, low in BCCs and MSCCs. (**a**,**b**) Microphotographs show representative images of immunohistochemical (IHC) staining of BCC or non-metastatic SCC (NMSCC) biopsies for (**a**) Cyclin E (CE) or (**b**) *γ*H2AX. See also Supplementary Figures 2a,b. Histograms show histoscore values for CE (**a**) or *γ*H2AX (**b**) in the series of BCCs (*n*=15) and SCCs (*n*=35). Scale bars, 200 μm. (**c**) Left histogram: histoscore values for CE in NMSSC (*n*=17) and metastatic SCC (MSCC; *n*=18). Right: bar histogram shows percent of cases displaying nuclear or cytoplasmic localisation of CE (nuclear or cytoplasmic) within NMSCCs (light grey) or MSCCs (dark grey). See also Supplementary Figure 2b. (**d**) Histoscore values of *γ*H2AX in NMSCC and MSCC (left) or in SCC well differentiated (Diff; *n*=10), moderately differentiated (Mod; *n*=20) and poorly differentiated (Poor; *n*=5) (right; see also Supplementary Figure 2a). Data plotted by tests Kruskal-Wallis/Mann-Whitney U. *P<0.05 and **P<0.01

The results above suggest that an excess of Cyclin E by inducing DNA damage and differentiation might be a burden to carcinoma cells. We studied the expression of Cyclin E and *γ*H2AX in a pilot collection of human BCC and SCC biopsies. 15 BCCs, 17 non-metastatic SCCs (NMSCCs) and 18 metastatic SCCs (MSCCs) were compared (Supplementary Table 1). Both Cyclin E and *γ*H2AX were barely detectable in BCC lesions, whereas they were prominent in SCC lesions (Figures 2a and b). *γ*H2AX in BCCs was detectable only in the growing front of the tumour (Figure 2b; Supplementary Figure 2a). Nuclear expression of Cyclin E was significantly lost in MSCCs as compared with NMSCC. In 40% of MSCCs Cyclin E was strongly accumulated in the cytoplasm (cCE; *P*<0,01; Figure 2c; Supplementary Figure 2b). Although the number of cases is small, cCE was found only in one case out of 16 NMSCCs and none of 10 well-differentiated SCCs. While the mechanism exporting Cyclin E to the cytoplasm and its consequences are unclear, overexpression of a cytoplasmic shorter form of the protein accelerates mitosis (low molecular weight LMW-Cyclin E).^23, 31^ Interestingly, we detected a shorter protein in SCC12F overexpressing Cyclin E (Figure 1b). Reducing Cyclin E in the nucleus by sequestering cdk2 in the cytoplasm might reduce DNA replication-associated damage. To note, the DNA damage marker *γ*H2AX was strong in NMSCCs and significantly lost in poorly differentiated MSCCs (Figure 2d; Supplementary Figure 2a). Analyses of serial sections showed coincident Cyclin E and *γ*H2AX in cases where Cyclin E was strongly nuclear and marked loss of *γ*H2AX in cases where it was strongly cytoplasmic (Supplementary Figure 3a). Co-accumulation of Cyclin E and p21 reveals cell cycle conflict, occurs in the onset of initiation of keratinocyte differentiation (Supplementary Figures 3b–d)^9, 20, 29, 30^ and was found in well differentiating SCCs displaying nuclear Cyclin E (Supplementary Figures 3c,d). p21 was strong in large nuclei or multinucleate cells (arrows in Supplementary Figure 3d). No general correlation was found between p21 or p53 and aggressiveness (Supplementary Figure 4a). However, deregulated p53 (maximum intensity in 100% of cells), suggestive of inactivating mutations, was found in 44% of cases with cCE *versus* only 13% with no cCE (Supplementary Figure 4b).

**Figure 3:**
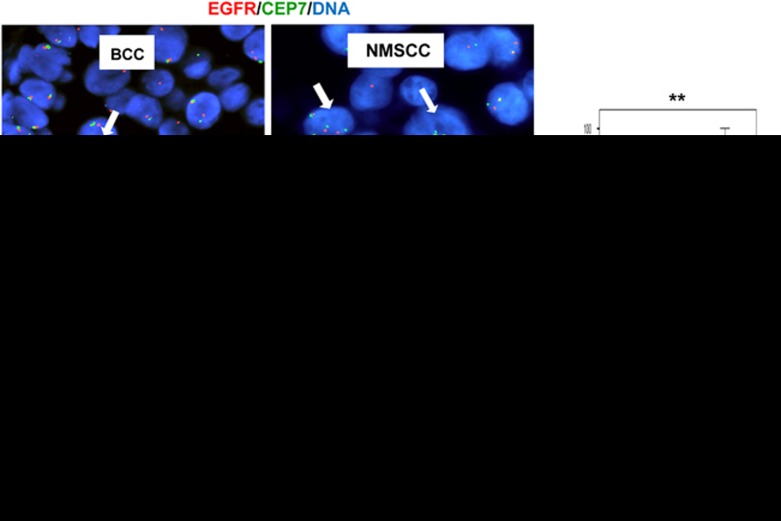
Chromosomal alterations are low in BCCs, moderate in NMSCCs and high in MSCCs. Representative microphotographs of top: *in situ* hybridisation (FISH) for the EGFR locus (red) and centromere of chromosome 7 (CEP7; green) in sections of BCC or non-metastatic SCC (NMSCC); bottom: NMSCC or metastatic SCC (MSCC) hybridised for EGFR (red). DAPI for DNA in blue. Scale bar, 25 μm. Histogram: percent of nuclei with EGFR amplifications (>3 spots) in BCC, NMSCC and MSCC (*n* as in Figure 2). Data plotted by tests Kruskal-Wallis/Mann-Whitney U. **P<0.01

The results above suggest that the axis squamous differentiation/Cyclin E via cell cycle stress might contribute to genomic instability in SCC. Consistently, MSCCs in the biopsy collection significantly displayed more chromosomal alterations than NMSCCs and these in turn more than BCCs (Figure 3). BCCs not showing signs of squamous differentiation, nor accumulation of Cyclin E, contained small and homogenous nuclei with two chromosomal copies.

**Figure 4:**
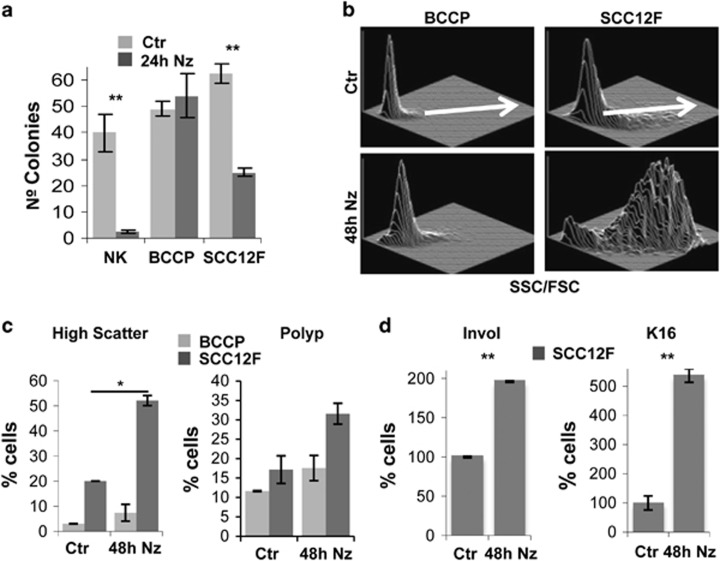
A Nocodazole treatment induces a partial anti-proliferative squamous differentiation response in SCC12F cells but not in BCCP cells. (**a**) Clonogenic capacity of normal keratinocytes (NK), BCCP and SCC12F untreated (DMSO only, Ctr) or after a 24h Nocodazole (Nz) treatment (Supplementary Figure 5a). (**b**) Representative 3D histograms of light scattering (SCC: Side Scatter; FSC: Forward Scatter; cell size and complexity; white arrow) in BCCP and SCC12F untreated (Ctr) or treated with Nz for 48 h. (**c**) Percent of cells with large size and complexity (High Scatter) or polyploidy (Polyp, DNA content >4N), as measured by flow-cytometry in BCCP (light grey) or SCC12F (dark grey) treated with Nz for 48 h or untreated (Ctr) as indicated. See also Supplementary Figures 5b–d. (**d**) Percent of SCC12F cells treated with Nz for 48 h that express the differentiation markers involucrin (Invol) or keratin K16, relative to untreated cells, as measured by flow-cytometry. Error bars are s.e.m. of duplicate or triplicate samples of at least two independent representative experiments.*P<0.05 and **P<0.01. See also Supplementary Figure 6

We investigated whether the capacity to escape the differentiation-associated cell division block in spite of genetic damage may cause genomic instability in SCC cells. To this end, we subjected BCCP and SCC12F cells to mitosis blocks by use of the microtubule-inhibitory drug Nocodazole (Nz), which triggers the squamous differentiation programme in human keratinocytes within 48 h^9, 14^ (Supplementary Figure 5). This response mimics differentiation induced by MYC, Cyclin E, loss of p53 or other inhibitors of mitosis.^9, 10, 20^ A 24 h Nz treatment irreversibly suppressed the clonogenic capacity of normal keratinocytes (Figure 4a; Supplementary Figure 5a). However, SCC12F conserved some of the capacity to proliferate after the mitosis block and the clonogenic capacity of BBCP cells was barely affected (Figure 4a; Supplementary Figure 5a). This suggests that BCCP cells have a more robust G2 arrest and a tighter control of cell growth. Accordingly, while SCC12F cells strikingly increased in cell size upon the mitosis block (high light scattering typical of differentiated keratinocytes),^32^ the size of BCCP cells changed very moderately (Figures 4b and c; Supplementary Figures 5c and d). In addition, SCC12F slipped into polyploidy at a greater extent than BCCP (Figure 4c; Supplementary Figures 5c and d). The changes in cellular size and ploidy in SCC12F were associated with an increase of squamous suprabasal markers (involucrin and keratin K16; Figure 4d; Supplementary Figures 6a and b), indicating that these cells conserve a partial differentiation response to mitotic stress. Differentiation likely accounts for the loss of clonogenicity observed, as no signs of apoptosis were found in the DNA content profiles (Supplementary Figures 5 b-d). In contrast, squamous markers were undetectable in BCC cells upon Nz treatment (Supplementary Figure 6b, not shown) in line with the absence of clonogenic loss.

Proliferating SCC12F cells just after the Nz drug release were larger and a high proportion polyploid. Therefore these cells were able to proliferate in spite of being polyploid (Supplementary Figures 7a and b). However, after two passages subculture these cells (SCC12R1) displayed higher levels of p53, p21 and DNA damage marker *γ*H2AX and were more proliferative than the parental cells (Supplementary Figures 7c–f). p53 was overexpressed in SCC12R1 and the typical mutated conformation was now detected, although the cell morphology was unchanged.

**Figure 5:**
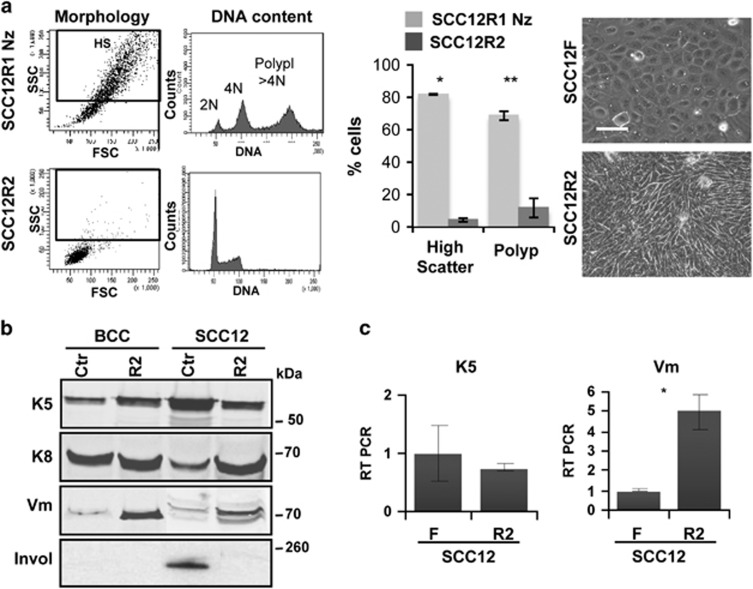
Two consecutive Nocodazole mitotic blocks drive a phenotypic conversion in SCC12F cells. (**a**)Left plots: representative flow-cytometry analyses of morphology (light scattering) and DNA content (propidium iodide) of SCC12R1 immediately after a second 48 h Nocodazole (Nz) treatment (SCC12R1Nz), or cells growing after treatment release (SCC12R2). Black square in dot plots gates cells with high light scatter (HS). Middle: bar histogram shows percent of SCC12R1Nz cells (light grey) or SCC12R2 cells (dark grey) with high scatter or polyploid (Polyp, DNA content >4N). Right: phase contrast microphotographs of parental SCC12F and SCC12R2 as indicated. Scale bar, 50 *μ*m. (**b**) Western blotting for the expression of keratin K5, keratin K8, vimentin (Vm) or involucrin (Invol) in *insoluble* extracts of untreated BCCP or SCC12F (Ctr), or after a second Nz treatment release (R2). Same number of cells per lane. See also Supplementary Figure 8b. Invol lanes for Ctr are the same as in Figure 1c. Uncropped blots are shown in Supplementary Figure 12. (**c**) Quantitation of the expression of keratin K5 or vimentin (Vm) in SCC12F and SCC12R2 by real-time (RT)-PCR. See also Supplementary Figure 8b. Error bars are s.e.m. of duplicate samples of at least two independent representative experiments. *P<0.05, **P<0.01

In order to test whether consecutive partial mitotic blocks might induce severe changes in SCC12F cells as it might occur *in vivo*, we subjected SCC12R1 cells to a second 48 h Nz treatment. Again, the mitotic block at first produced a high proportion of polyploid cells and significantly reduced their clonogenic potential (SCC12R1Nz; Figure 5a; Supplementary Figure 8a). However, cells stably growing two passages after the second block (SCC12R2) displayed a markedly different phenotype. They were remarkably homogenous in size, small and diploid and displayed a more fibroblastic morphology (Figure 5a). To study this phenomenon further we performed western blotting, immunofluorescence or RT-PCR for expression of keratin K5, often lost in aggressive carcinomas, keratin K8 and vimentin, often gained in aggressive carcinomas. Interestingly, SCC12R2 lost expression of epidermal keratin K5 and Cyclin E and strongly gained keratin K8 and vimentin with respect to untreated parental cells (Figures 5b and c; Supplementary Figure 8b). Consequently, SCC12R2 cells lost completely the expression of the typical squamous marker involucrin (Figure 5b).

Similar phenotypic results were obtained on R2 cells in three independent experiments and the degree of the changes was proportional to the number of consecutive mitotic blocks applied (R1, R2, R3; Supplementary Figure 9). Altogether the results indicate that the phenotypic changes were not sporadic events but a consistent conversion produced by mitotic stress.

**Figure 6:**
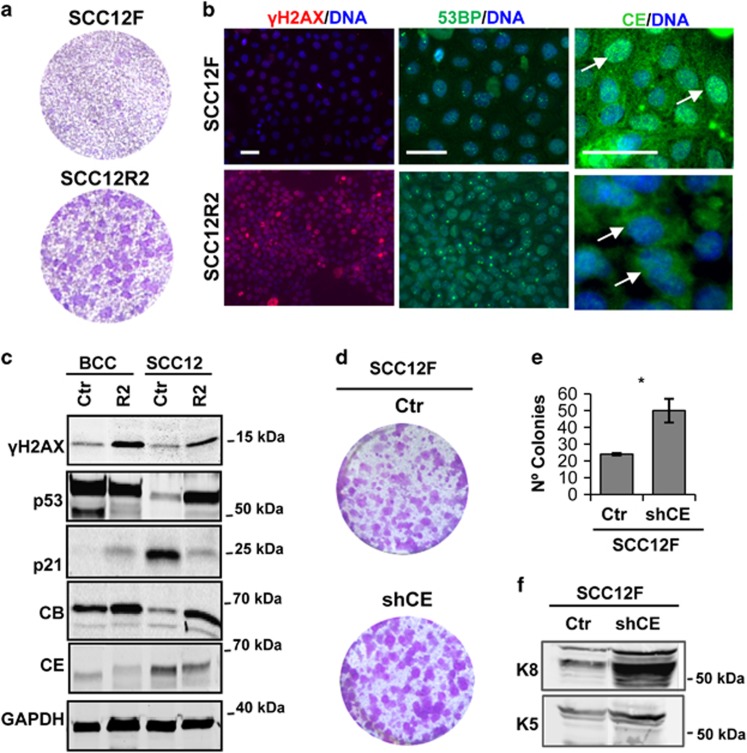
SCC12R2 or SCC12F expressing shRNA to Cyclin E display growth advantage. (**a**) Clonogenic capacity of parental SCC12F and SCC12R2 (1 000 cells per well). Wells representative of tripliates of three independent experiments. (**b**) Immunofluorescence for *γ*H2AX (red; left panels), 53 binding protein 1 (53BP, green; middle panels) or Cyclin E (CE, green; right panels) in SCC12F or SCC12R2. DAPI for DNA in blue. Scale bar, 50 *μ*m. Note that reduced Cyclin E, prominent and nuclear in SCC12F, localises in the cytoplasm in SCC12R2 (arrows). (**c**) Western blot for expression of *γ*H2AX, p53, p21, Cyclin E (CE) or Cyclin B (CB) in untreated BCCP and SCC12F (Ctr), or in growing cells after a second Nz treatment (R2). GAPDH is loading control. Lanes for Ctr are the same as in Figure 1b. (**d**) Clonogenic capacity of SCC12F infected with the empty vector (Ctr) or with specific shRNA to Cyclin E (shCE; 2 500 cells per well). (**e**) Bar histogram shows the number of proliferative colonies in (**d**). Error bars are s.e.m. of triplicate samples. *P<0.05. (**f**) Western blotting for expression of keratin K5, keratin K8 in Ctr and shCE. See also Supplementary Figure 10. Uncropped images of blots in **c** and **f** are shown in Supplementary Figure 12. In (**a** and **d**) wells are representative of triplicate samples

The proliferative capacity of SCC12R2 cells was significantly enhanced compared with parental cells (Figure 6a), in spite of a neat accumulation of *γ*H2AX (Figure 6b). Western blot analyses confirmed the higher levels of *γ*H2AX in SCC12R2 (Figure 6c). The expression of the DNA repair factor p53-binding protein 1 (53BP) was low in BCCP cells and high in SCC12F cells (Figure 6b; Supplementary Figure 8c). Interestingly, SCC12FR2 cells displayed a strong nuclear spot of 53BP typical of isolated unrepaired DNA damage.^33^ In addition, SCC12R2 strongly expressed the mutated p53 antigen Pab240 (p53mut; Supplementary Figure 8b) and strongly accumulated p53 (Figure 6c), indicative of inactivating mutations.^27^ Proliferating SCC12R2 cells also lost p21, decreased the expression of Cyclin E and increased the expression of mitotic Cyclin B, resembling BCCP cells (Figures 6b and c). Interestingly, SCC12R2 accumulated Cyclin E in the cytoplasm. These changes were consistent with the loss of the squamous phenotype, nuclear Cyclin E and *γ*H2AX observed in aggressive MSCCs *in situ*.

In order to test whether loss of Cyclin E function might contribute to the loss of the squamous phenotype, we inhibited the endogenous protein in well-differentiated parental SCC12F. To this aim, we made use of lentiviral constructs carrying specific shRNA to Cyclin E (shCE). Interestingly, shCE diminished the levels of *γ*H2AX, potentiated the expansion of SCC12F colonies, induced expression of keratin K8 and vimentin and produced a more fibroblastic morphology (Figures 6d–f; Supplementary Figure 10).

**Figure 7:**
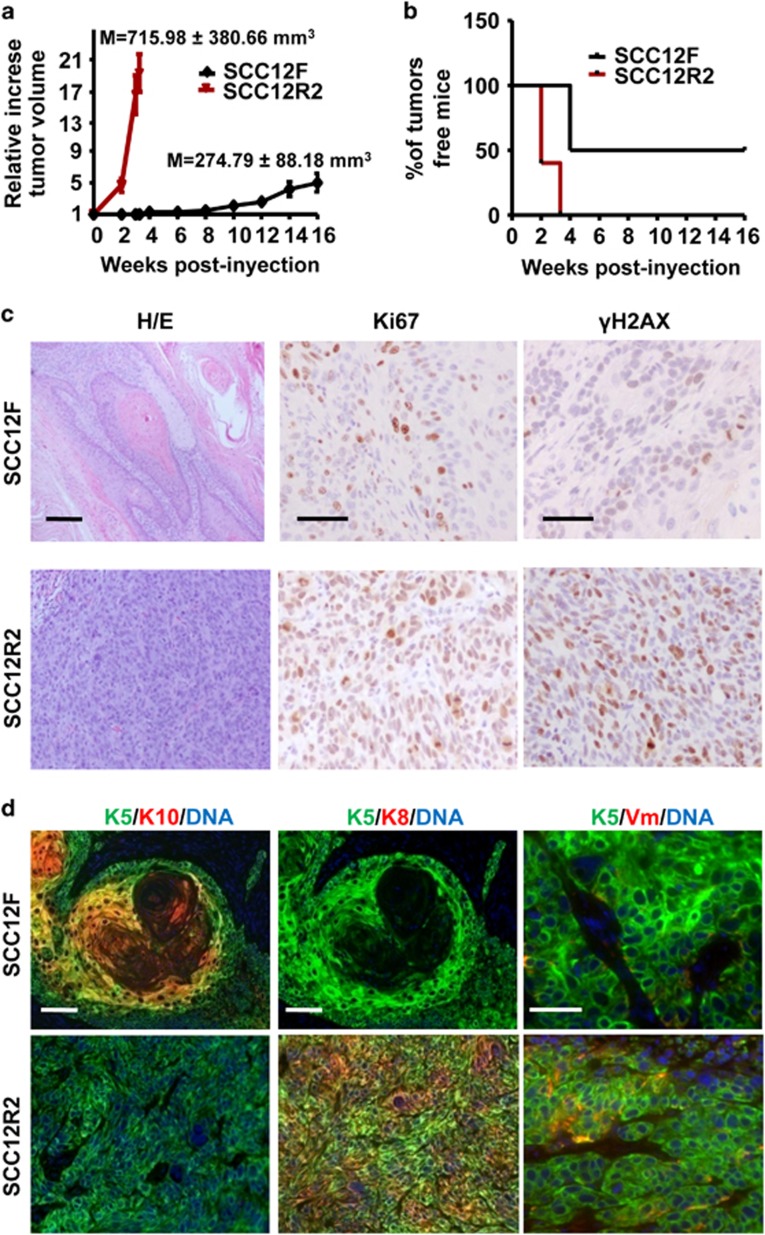
Two consecutive Nocodazole mitotic blocks drive squamous malignant progression. Plots for the tumorigenic capacity of parental SCC12F (black) or SCC12R2 (red) in nude mice. (**a**) Growth rate of tumours with time; means of final volumes are indicated (M). (**b**) Number of mice with no tumours with time. (**c**) Hematoxylin-eosin staining or immunohistochemistry for Ki67 and *γ*H2AX of microsections of tumours generated by SCC12F or SCC12R2, as indicated. (**d**) Immunofluorescence for keratin K5 (green) and keratin K10 (red; left panels), K5 (green) and keratin K8 (red; middle panels) or K5 (green) and vimentin (red, Vm; right panels) of SCC12F or SCC12R2 tumours, as indicated. See also Supplementary Figure 11. DAPI for DNA in blue. Scale bars, 200 *μ*m

To determine whether the double mitotic block rendered SCC cells more malignant, we injected SCC12R2 subcutaneously in nude mice. Parental SCC12F are scarcely tumorigenic.^25, 26^ As shown in Figure 7a, the parental cells developed very slowly growing benign tumours that were detected only after 10 weeks. In striking contrast, SCC12R2 cells rapidly generated detectable tumours after only 10 days. The size of the SCC12R2 lesions was also larger at term. Half of the mice injected with the parental cells never developed tumours after 16 weeks, whereas all mice injected with SCC12R2 cells had developed tumours after 4 weeks (Figure 7b). The histology showed that the tumours generated by parental cells were well-differentiated, whereas those generated by SCC12R2 cells were characterised as poorly differentiated (H/E; Figure 7c). SCC12R2 tumours were more proliferative (Ki67) and accumulated *γ*H2AX (Figure 7c). Consistently, these rapidly growing tumours displayed loss of epidermal keratins K5/K10 and E-cadherin and gain of keratins K13, K8 and vimentin (Figure 7d; Supplementary Figure 11), changes typical of aggressive stages of skin carcinogenesis.^34, 35^ The abundance of *γ*H2AX and the absence of lung metastasis (not shown) suggest that they are in an early stage of invasive conversion.

## Discussion

**Figure 8:**
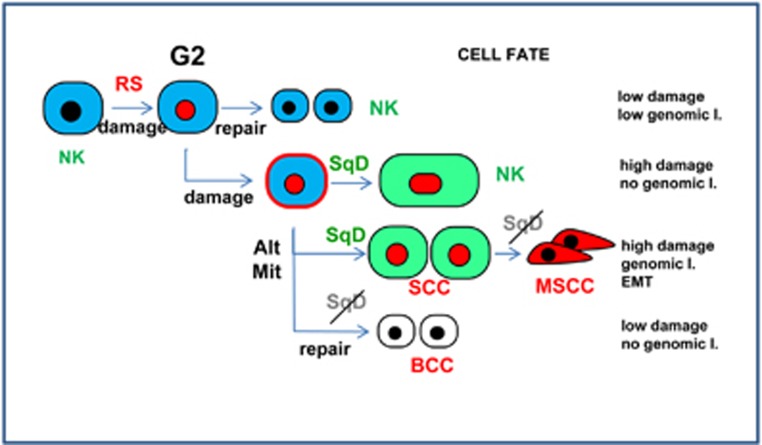
Model for the contribution of the squamous DNA damage-differentiation response to carcinogenesis. Normal keratinocytes (NK) accumulating Cyclin E and DNA damage due to cell cycle or mitotic stress (red nuclei), arrest in G2 for DNA repair. Repaired cells are allowed to divide. Cells with irreparable damage undergo mitotic bypass or mitotic slippage, increase of cellular size and squamous differentiation (SqD; Green), irreversibly unable to divide. Squamous carcinoma cells (SCC) with alterations in mitosis control (AltMit) are able to divide in spite of irreparable damage and polyploidy. Sustained stress and genomic instability (genomic I.) lead to further alterations giving rise to cells that lose the squamous phenotype, are able to divide in spite of numerous genetic alterations and eventually become metastatic (MSCC; dark blue). Basal cell carcinoma cells having alterations in mitosis control (AltMit) due to complete loss of the squamous pathway do not undergo mitotic bypass or slippage after G2 arrest with the possibility to repair and continue to divide normally with low genomic instability (BCC)

We hypothesised that alterations in the link between mitosis control and squamous differentiation might contribute to carcinogenesis.^10^ It is paradoxical that BCC of the skin is very rarely invasive in spite of losing the squamous phenotype. In our study SCC, not BCC cells, responded to cell cycle stress by initiating squamous differentiation. Cell cycle deregulation/replication stress induces DNA damage and G2/M arrest.^18, 19^ Moreover, mitotic arrest is sufficient to cause DNA damage^36^ and mitotic slippage/bypass can result in chromosomal defects.^10^ Moreover, polyploidy often leads to aneuploidy when cells are able to divide.^37^ We propose that the reversibility of the cell division block imposed by the initiation of squamous differentiation contributes to genomic instability and malignant progression in SCCs but not in BCCs (Figure 8).

Normal keratinocytes that initiate differentiation accumulate high levels of DNA damage marker *γ*H2AX,^10^ as we found in NMSCC cells. In contrast, MSCCs lost nuclear Cyclin E and the *γ*H2AX signal. Accumulation and coexpression of Cyclin E and *γ*H2AX in NMSCCs and their coincident nuclear loss in MSCC suggests that high levels of nuclear Cyclin E via replication stress^9, 22^ is a burden to malignant carcinoma cells. While normal keratinocytes differentiate terminally in response to cell cycle stress,^10, 11^ damaged SCC cells that are able to divide are genetically instable. This would increase the probability of more aggressive clones to appear that would be selected for (Figure 8). Interestingly, genomic instability has been shown to promote evolutionary adaptation.^38^ In our study simply overexpressing Cyclin E was insufficient to drive malignant transformation. However, forcing cell division by FOXM1 allows damaged normal keratinocytes with deregulated MYC or p53 to amplify.^21^ FOXM1 is a global mitotic regulator and this suggests that mitosis control is a limiting factor in keratinocyte transformation. Squamous cancer progression might require mitotic alterations.

Remarkably, in our study two consecutive mitotic blocks were sufficient to render well-differentiated SCC cells highly tumorigenic. These cells initiated an epithelial–mesenchymal conversion typical of invasive SCCs^34, 35, 39^ and proportional to the number of consecutive mitotic blocks (SCC12R1, R2, R3). The phenotypic conversion of SCC12R2 cells, far more aggressive than the parental cells, shows that mitotic stress can contribute to squamous malignant progression. Paradoxically, the SCC12FR2 cells shared features with BCCP cells. As in carcinoma biopsies, both lines displayed low levels of *γ*H2AX. The critical difference might be the degree of genetic alterations caused by genomic instability. Accordingly, MSCCs displayed a higher degree of chromosomal alterations and SCC12R2 cells strongly displayed spots of 53BP, a marker of persistent unrepaired DNA damage.^33^ In contrast, no spots 53BP were observed in BCCP cells. The higher stability of the BCC genome might be due to the lack of the squamous pathway and, a more robust G2 arrest and a tighter control of cellular growth. In contrast, SCC12F cells displayed a loose mitotic control. Interestingly, mutations in the Sonic hedgehog (Hh) pathway are frequent in BCC, suppress squamous differentiation in mouse^40^ and cause evasion of G2/M checkpoints.^41^ The robustness of the G2/M arrest in BCC cells might allow more efficient DNA repair and maintenance of genomic stability. Consistently, *γ*H2AX in BCC biopsies was scarce while it was detected in the tumour growing front and in proliferating BCCP *in vitro*.

The epithelial–mesenchymal conversion has recently been associated with loss of *γ*H2AX.^42^ The axis squamous differentiation/high Cyclin E constitutes a mitotic barrier^11^ and we argue that the most aggressive SCC cells lose the squamous phenotype in order to avoid it. This is well supported by the loss, or cytoplasmic accumulation of Cyclin E (cCE) that we found in metastatic SCCs in our pilot study and in SCC12R2 *in vitro*. Recent works on large biopsy collections also have shown association between cytoplasmic shorter forms of Cyclin E and poor breast cancer prognosis,^23, 31, 43^ suggesting a growth advantage to cancer cells. Although cCE can cause genomic instability, it accelerates mitosis.^23, 31^ In our study, the loss of *γ*H2AX was especially marked in cases displaying cCE. By reducing the levels of Cyclin E in the nucleus, malignant squamous cells might in part avoid severe deregulation of DNA replication-S phase. Functionally supporting this model, in our study inhibition of Cyclin E in well-differentiated SCC12F reduced DNA damage and enhanced proliferation and expression of mesenchymal markers.

Loss of p53, *The Guardian of the Genome,* leads to polyploidy in a variety of cell types.^44^ In keratinocytes this loss induces polyploidy and squamous differentiation.^10^ The responses of the carcinoma cells studied here do not seem to be mediated by p53: (i) SCC12F cells seemingly bearing intact p53 become polyploid upon Nocodazole; (ii) BCCP displaying mutated p53 were able to efficiently control G2/M and ploidy; (iii) SCC12R2 cells overexpressing mutated p53 displayed no signs of polyploidy. In addition, the levels of p53 in the human biopsies did not indicate a general association with aggressiveness. However, we detected a potential association between cCE and deregulation of p53. In addition, the cell cycle inhibitor p21, target of p53, stayed high in MSCC. Interestingly, Galanos *et al.* now reports a role of chronic and p53-independent expression of p21 in promoting genomic instability through replication stress in carcinomas of lung of head and neck.^45^ Moreover, the deregulation of DNA replication licensing protein cdc6 contributes to features of epithelial–mesenchymal transition^46^ and deregulated Cyclin E was found to affect licensing.^47^

In summary, our model is that the DNA damage-squamous differentiation pathway constitutes first a barrier to undesired proliferation, second a source of genomic instability, thereby driving malignant progression of genetically damaged cells that are able to divide (Figure 8). The loss of detectable nuclear Cyclin E and *γ*H2AX in MSCCs in the pilot series of biopsies studied was highly significant. These results, together with the cytoplasmic accumulation of Cyclin E, should encourage further studies on larger cohorts of squamous carcinomas. The findings might have application into squamous cancer in locations other than skin, as a growing body of evidence suggests that they might share common mechanisms. It has been proposed that alterations in ploidy contribute to cancer malignancy.^37^ Our observations would indicate that squamous cancer cells become malignant not because they are polyploid, but because they are capable to divide in spite of being so.

## Materials and methods

### Cell culture, human biological samples and viral infections

Ethical permission for this study was requested, approved, and obtained from the Ethical Committee for Clinical Research of Cantabria Council, Spain. In all cases of primary cell culture, human tissue material discarded after surgery was obtained with written consent presented by clinicians to the patients and it was treated anonymously.

The Basal carcinoma cell line (BCCP^24^), isolated from a human facial BCC, was kindly provided by Dr. R. Polakowska (Institut pour la Recherche sur le Cancer de Lille [IRCL], Lille Cedex, France). The SCC12F line was cultured from a human facial SCC.^25^ SCC12B originated from a more aggressive component of the same carcinoma was also analysed. Primary normal keratinocytes (NK) were isolated from neonatal human foreskin. All cells were cultured in presence of a mouse fibroblast feeder layer (inactivated by mitomycin C) in Rheinwald FAD medium as described previously (10% serum and 1.2 mM Ca^+2^).^48^ All cell lines and primary normal keratinocytes used were tested for mycoplasma contamination.

Cells were treated with Nocodazole for 24 h (Nz; 20 *μ*M; Sigma-Aldrich, St. Louis, MO, USA).^49^ Primary normal keratinocytes were treated 24 h with doxorubicin (0.5 *μ*M). Parallel control cultures were always subjected to the DMSO vehicle only. Fresh medium was added 24 h before addition of Nz and again 24 h after.

BCCP or SCC12F were subjected to retroviral infection with pBabe-GFP (GFP) and pBabe-GFP-Cyclin E (CEGFP) constructs. SCC12F also were subjected to lentiviral infection as described (see also Supplementary Materials and Methods)^9, 10^ with MISSION Sigma-Aldrich plasmids: control (pLKO.1, Ctr) and plKO.1 with a shRNA against Cyclin E (TRCN0000045300, shCE). The studies were carried out either by analysing unselected pools 4 days after retroviral infections or by stably selecting cells expressing retroviral or lentiviral constructions by 1*μ*g/ml puromycin.

Clonogenicity assays were made as described previously (see also Supplementary Materials and Methods).^10^

50 non-melanoma skin cancer lesions were included in the study (Supplementary Table 1): (i) 15 basal cell carcinomas; (ii) 18 primary metastatic squamous cell carcinomas (MSCCs) that had evolved to (histologically confirmed) lymph node metastases (2 well differentiated; 11 moderately differentiated; 5 undifferentiated) and (iii) 17 patients with SCC who had not developed any metastasis (non-metastatic, NMSCCs) in a 5-year follow-up period (8 well differentiated; 9 moderately differentiated; 0 undifferentiated). A control group of 10 samples of elastotic non-tumoral skin was also included in the study. For details of sample collection and characterisation see Supplementary Materials and Methods. After histopathological evaluation the invasive edge of the tumour was selected for the construction of tissue microarray (TMAs).^50^ Two tissue cylinders with a diameter of 2 mm were punched from the selected areas from each tissue block and brought into a recipient paraffin block using the tissue micro-arrayer (Arrayer Punch set 2.00 mm, ATA200, Advanced Tissue Arrayer, Chemicon International).

### Antibodies

Primary and secondary antibodies utilised in this study are listed in Supplementary Materials and Methods.

### Flow-cytometry

Cells were harvested, fixed and stained as previously described for DNA synthesis and content (BrdU incorporation and propidium iodide; see Supplementary Materials and Methods).^9, 14^

### Histology and immunostaining

For immunofluorescence, cells were grown on glass coverslips, fixed, and stained as previously described.^9^ H&E, immunofluorescence and immunohistochemical stainings of carcinoma sections of paraffin embedded formalin fixed tissues were performed on 4*μ*m thick sections. For more details see Supplementary Materials and Methods.

The score of immunohistochemical (IHC) stainings was given between 0 and 100% of positively stained neoplastic cells and the intensity was measured as: 1 (weak), 2 (medium), 3 (strong). The whole punch (2 mm in diameter, 3.14 mm^2^) was evaluated using a 10 × objective lens and 10 × ocular lens. The histoscore was calculated by multiplying the percent of positive cells by the intensity (from 0 to 3) to give numbers ranging from 0 to 300. p53 was considered deregulated by inactivating mutations when the intensity of the staining was 3 (maximum) in 100% of cells (histoscore 300). Histograms were then plotted using Test of Kruskal–Wallis in combination with Mann–Whitney U test. Immunohistochemical staining was independently evaluated by two observers (A. Toll and D. López).

For analyses of protein expression, cells were washed with PBS, lysed and subjected to SDS-PAGE electrophoresis and western blotting as described.^9^ *Insoluble* protein fractions were incubated in Urea lysis buffer (10 mM Tris pH 8, 5% SDS, 5% *β*-mercaptoethanol, 4 M Urea). The whole original blots are shown in Supplementary Figure 12.

### RT-PCR

Total RNA was isolated using NucleoSpin RNA (Macherey-Nagel, Düren, Germany) and reverse transcribed with the iScript cDNA synthesis kit (Bio-Rad, Hercules, CA, USA).^10^ The cDNAs (1 *μ*l) were amplified by real-time PCR (Bio-Rad iQ SYBR green supermix) and normalised to *β*-actin mRNA levels.^10^ Primers utilised in this study are listed in Supplementary Materials and Methods.

### Fluorescence *in situ* hybridisation

To evaluate genomic instability, fluorescence *in situ* hybridisation (FISH) with a specific probe against EGFR was also performed. Dual-colour hybridisation with fluorescent DNA for the centromeric region of chromosome 7 (CEP7, green) and for the specific DNA region for EGFR (7p12, red) was performed (Abbott Molecular, Abbot Park, IL, USA). One hundred nuclei per case were scored to determine the percent of epithelial cells with EGFR gains (three or more signals for EGFR). FISH were evaluated by two observers (A. Toll and D. López).

### Tumorigenesis

Experiments using animals were performed in compliance with the United Kingdom Coordinating Committee on Cancer Prevention Research's Guidelines for the Welfare of Animals in Experimental Neoplasia, and authorised by the Consejería de Medioambiente y Ordenación del Territorio de la Comunidad de Madrid. Further details on mice conditions in Supplementary Materials and Methods

Keratinocytes were tripsinized and resuspended in a mixture (2:1) of PBS and Matrigel (BD Biosciences, San Jose CA, USA). A volume of 150 *μ*l of this suspension containing 1 × 10^6^ cells was subcutaneously inoculated into the right flank of each mouse. Tumour width (*W*) and length (*L*) were measured twice a week using an external caliper. Tumour volume was calculated using the formula 0.5 × *L* × *W*^2^ (ref. 51).

### Statistical analyses

For cell culture experiments standard deviation and variance were calculated and served as estimates of variation within each group of data. For statistical comparison of groups with similar variance, a homoscedastic *t*-test was performed. For statistical comparison of groups with diverging variance, a heteroscedastic *t*-test was applied. Data sets were compared using an unpaired Student’s *t*-test. A *P*-value of less than 0.05 was considered statistically significant. For histology and FISH statistical analyses were performed using Windows Statistical Package for Social Sciences version 17 (SPSS, Chicago, IL, USA). The non-parametric Mann–Whitney U test was used to compare the histoscore of different immunohistochemical markers and EGFR gains by FISH. For contingency tables, the Fisher exact test was used to assess the level of significance. In all cases, a 2-tailed *P*<0.05 was required for statistical significance. Data were plotted and analysed using Test of Kruskal–Wallis in combination with Mann–Whitney U test.
