# Supplementary material for: Inefficient differentiation response to cell cycle stress leads to genomic instability and malignant progression of squamous carcinoma cells
Source: Cell Death Dis. 2017 Jun 29;8(6):e2901–. doi: 10.1038/cddis.2017.259 (PMC5520915; doi:10.1038/cddis.2017.259)
Supplement: Supplementary Tables and Figures [file cddis2017259x1.ppt]

## Slide 1
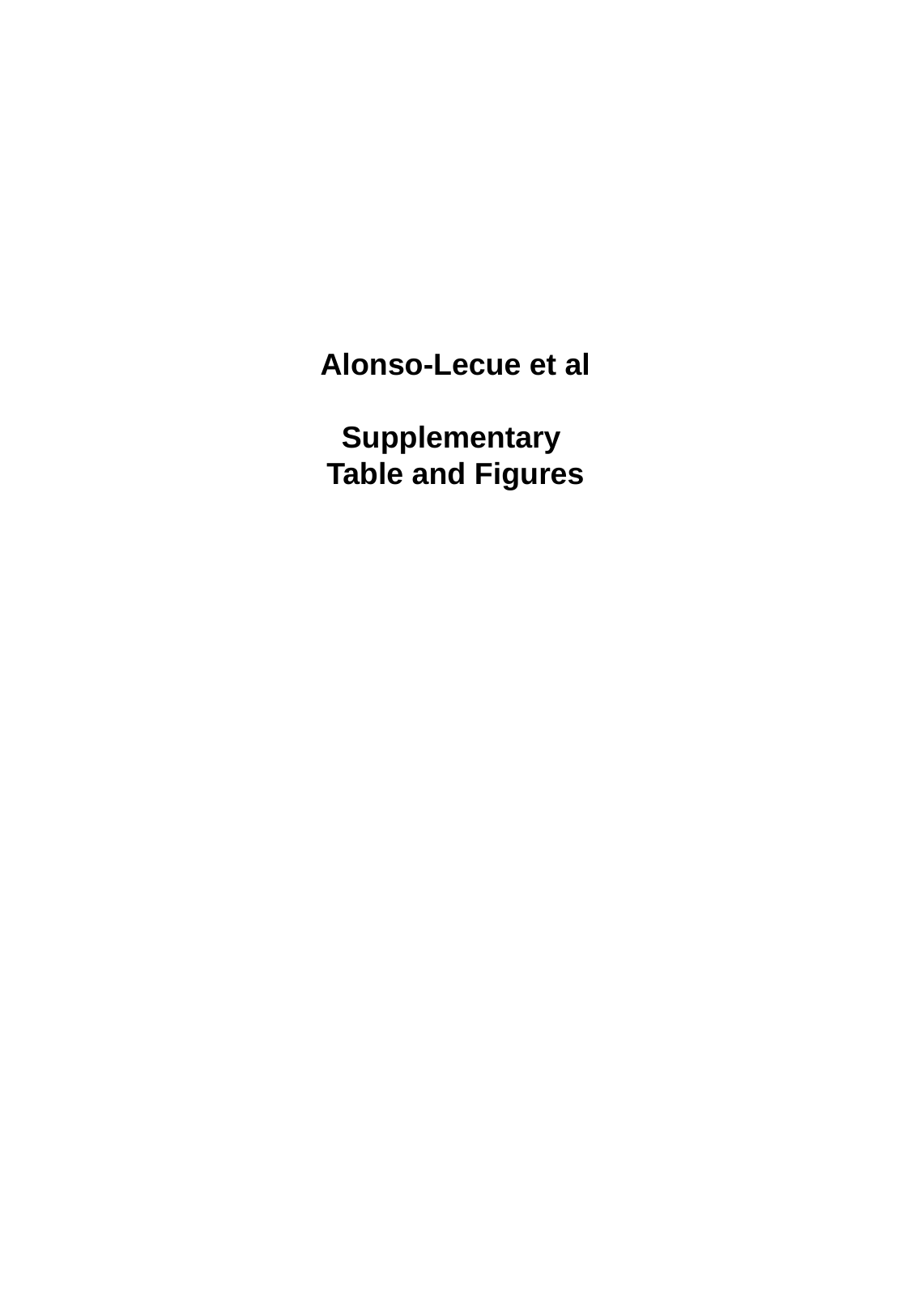

Alonso-Lecue et al
Supplementary
Table and Figures

## Slide 2
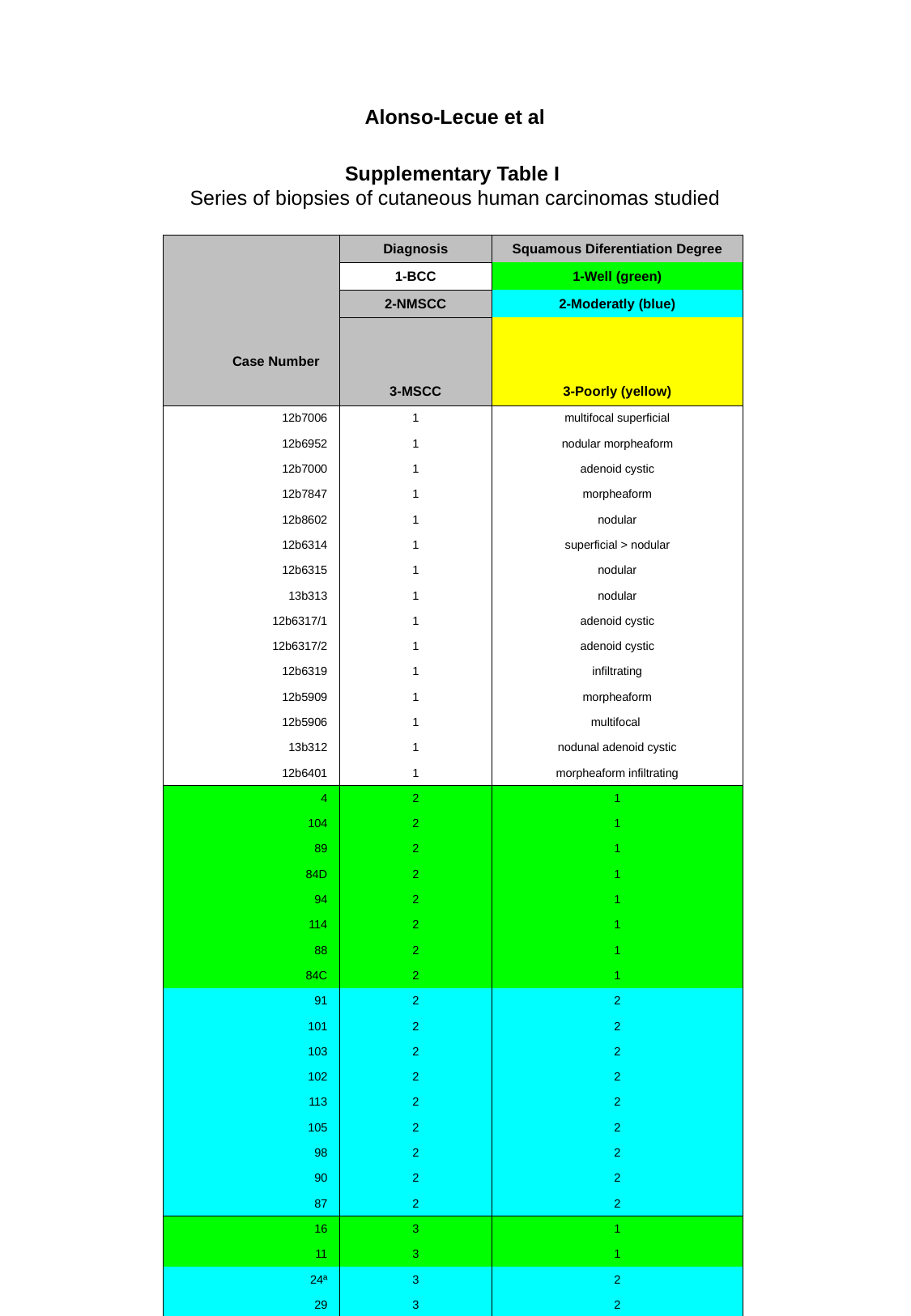

Alonso-Lecue et al
Supplementary Table I
Series of biopsies of cutaneous human carcinomas studied
| Case Number | Diagnosis | Squamous Diferentiation Degree |
| --- | --- | --- |
| | 1-BCC | 1-Well (green) |
| | 2-NMSCC | 2-Moderatly (blue) |
| | 3-MSCC | 3-Poorly (yellow) |
| 12b7006 | 1 | multifocal superficial |
| 12b6952 | 1 | nodular morpheaform |
| 12b7000 | 1 | adenoid cystic |
| 12b7847 | 1 | morpheaform |
| 12b8602 | 1 | nodular |
| 12b6314 | 1 | superficial > nodular |
| 12b6315 | 1 | nodular |
| 13b313 | 1 | nodular |
| 12b6317/1 | 1 | adenoid cystic |
| 12b6317/2 | 1 | adenoid cystic |
| 12b6319 | 1 | infiltrating |
| 12b5909 | 1 | morpheaform |
| 12b5906 | 1 | multifocal |
| 13b312 | 1 | nodunal adenoid cystic |
| 12b6401 | 1 | morpheaform infiltrating |
| 4 | 2 | 1 |
| 104 | 2 | 1 |
| 89 | 2 | 1 |
| 84D | 2 | 1 |
| 94 | 2 | 1 |
| 114 | 2 | 1 |
| 88 | 2 | 1 |
| 84C | 2 | 1 |
| 91 | 2 | 2 |
| 101 | 2 | 2 |
| 103 | 2 | 2 |
| 102 | 2 | 2 |
| 113 | 2 | 2 |
| 105 | 2 | 2 |
| 98 | 2 | 2 |
| 90 | 2 | 2 |
| 87 | 2 | 2 |
| 16 | 3 | 1 |
| 11 | 3 | 1 |
| 24ª | 3 | 2 |
| 29 | 3 | 2 |
| 1ª | 3 | 2 |
| 5B | 3 | 2 |
| 35 | 3 | 2 |
| 36 | 3 | 2 |
| 3 | 3 | 2 |
| 14 | 3 | 2 |
| 46ª | 3 | 2 |
| 7 | 3 | 2 |
| 5B | 3 | 2 |
| 12 | 3 | 3 |
| 31ª | 3 | 3 |
| 9 | 3 | 3 |
| 20 | 3 | 3 |
| 27 | 3 | 3 |

## Slide 3
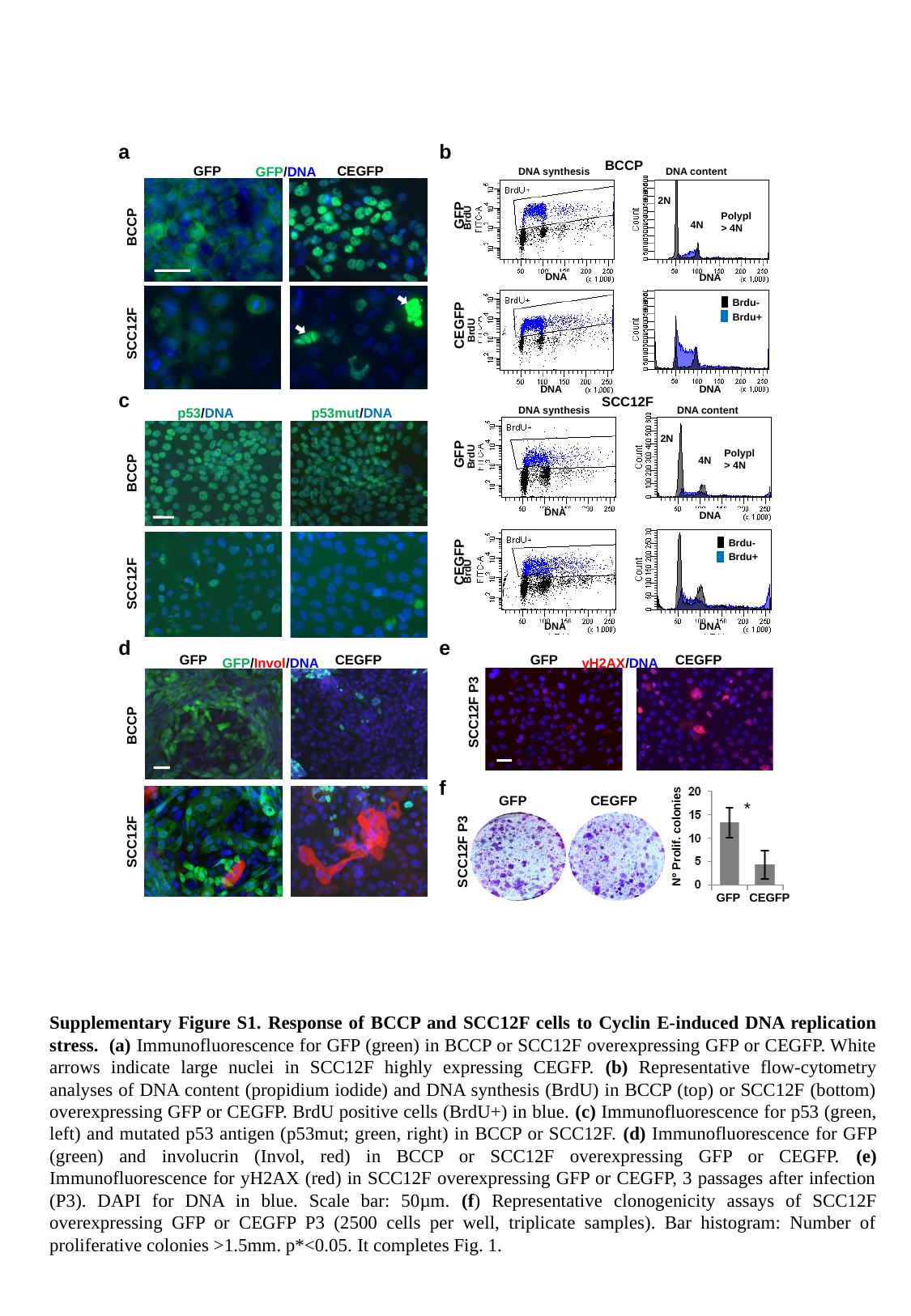

a
b
BCCP
DNA synthesis
DNA content
2N
GFP
Polypl
> 4N
BrdU
4N
DNA
DNA
Brdu-
Brdu+
CEGFP
BrdU
DNA
DNA
SCC12F
DNA synthesis
DNA content
GFP
2N
Polypl
> 4N
BrdU
4N
DNA
DNA
Brdu-
Brdu+
CEGFP
BrdU
DNA
DNA
GFP
CEGFP
GFP/DNA
BCCP
SCC12F
c
p53/DNA
p53mut/DNA
BCCP
SCC12F
d
e
GFP
CEGFP
GFP
CEGFP
GFP/Invol/DNA
γH2AX/DNA
SCC12F P3
BCCP
f
GFP
CEGFP
*
SCC12F
Nº Prolif. colonies
SCC12F P3
GFP
CEGFP
Supplementary Figure S1. Response of BCCP and SCC12F cells to Cyclin E-induced DNA replication stress. (a) Immunofluorescence for GFP (green) in BCCP or SCC12F overexpressing GFP or CEGFP. White arrows indicate large nuclei in SCC12F highly expressing CEGFP. (b) Representative flow-cytometry analyses of DNA content (propidium iodide) and DNA synthesis (BrdU) in BCCP (top) or SCC12F (bottom) overexpressing GFP or CEGFP. BrdU positive cells (BrdU+) in blue. (c) Immunofluorescence for p53 (green, left) and mutated p53 antigen (p53mut; green, right) in BCCP or SCC12F. (d) Immunofluorescence for GFP (green) and involucrin (Invol, red) in BCCP or SCC12F overexpressing GFP or CEGFP. (e) Immunofluorescence for yH2AX (red) in SCC12F overexpressing GFP or CEGFP, 3 passages after infection (P3). DAPI for DNA in blue. Scale bar: 50µm. (f) Representative clonogenicity assays of SCC12F overexpressing GFP or CEGFP P3 (2500 cells per well, triplicate samples). Bar histogram: Number of proliferative colonies >1.5mm. p*<0.05. It completes Fig. 1.

## Slide 4
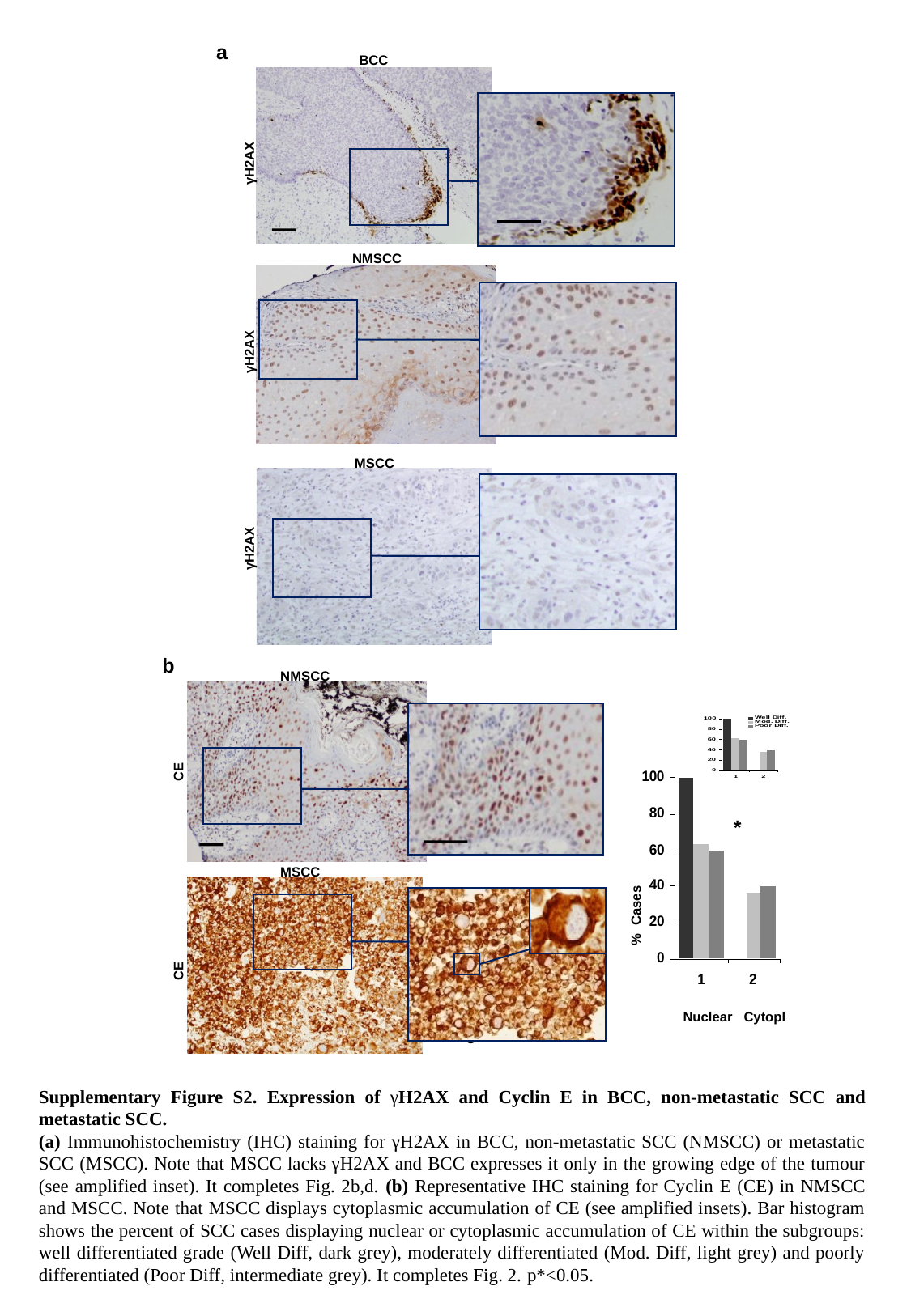

a
BCC
γH2AX
NMSCC
γH2AX
MSCC
γH2AX
b
NMSCC
CE
*
% Cases
MSCC
CE
Nuclear
Cytopl
MSCC
Supplementary Figure S2. Expression of γH2AX and Cyclin E in BCC, non-metastatic SCC and metastatic SCC.
(a) Immunohistochemistry (IHC) staining for γH2AX in BCC, non-metastatic SCC (NMSCC) or metastatic SCC (MSCC). Note that MSCC lacks γH2AX and BCC expresses it only in the growing edge of the tumour (see amplified inset). It completes Fig. 2b,d. (b) Representative IHC staining for Cyclin E (CE) in NMSCC and MSCC. Note that MSCC displays cytoplasmic accumulation of CE (see amplified insets). Bar histogram shows the percent of SCC cases displaying nuclear or cytoplasmic accumulation of CE within the subgroups: well differentiated grade (Well Diff, dark grey), moderately differentiated (Mod. Diff, light grey) and poorly differentiated (Poor Diff, intermediate grey). It completes Fig. 2. p*<0.05.

## Slide 5
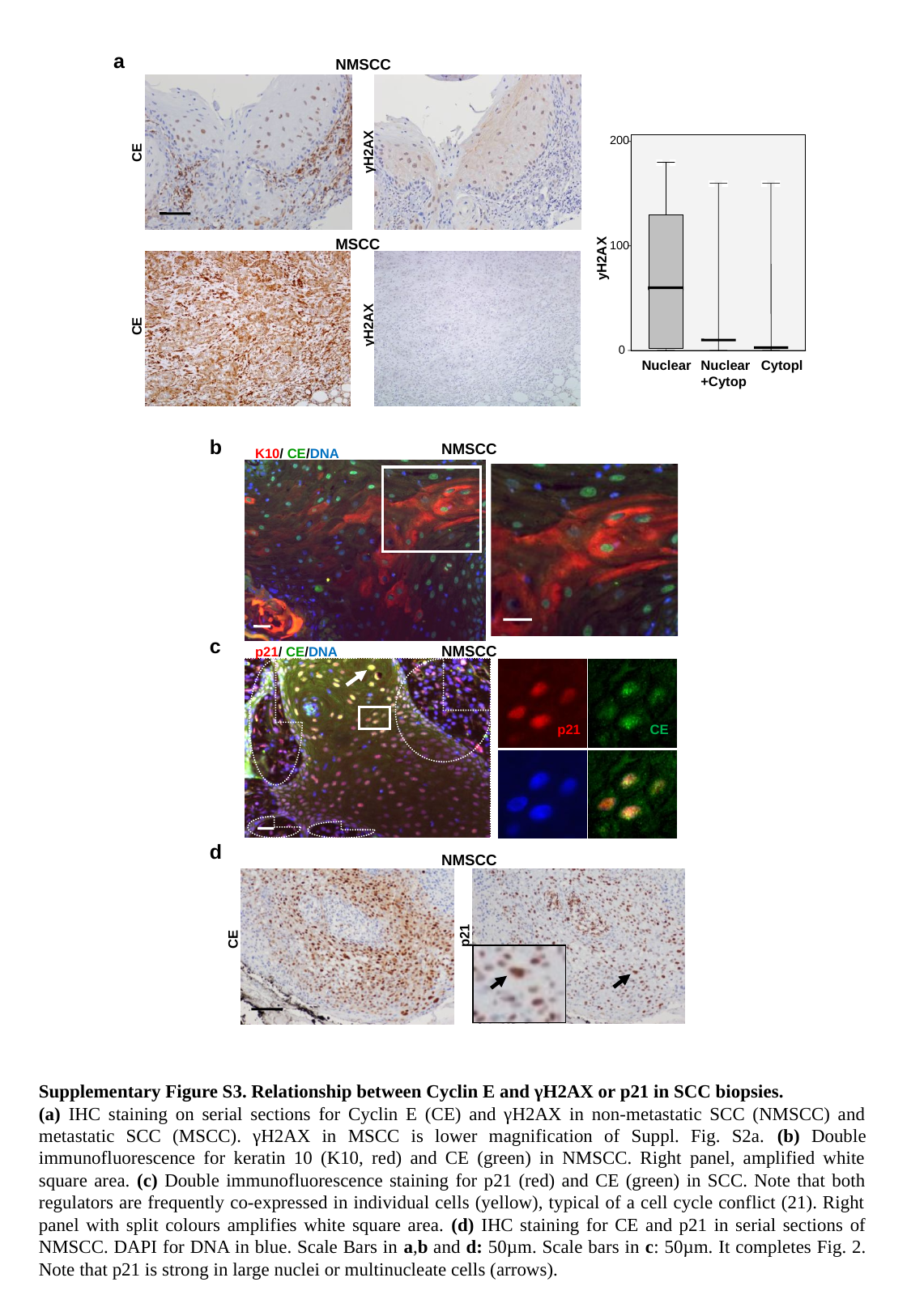

a
NMSCC
γH2AX
CE
200
MSCC
100
yH2AX
γH2AX
CE
0
Nuclear
Nuclear
+Cytop
 Cytopl
b
NMSCC
K10/ CE/DNA
c
NMSCC
p21/ CE/DNA
p21
CE
d
NMSCC
p21
CE
Supplementary Figure S3. Relationship between Cyclin E and γH2AX or p21 in SCC biopsies.
(a) IHC staining on serial sections for Cyclin E (CE) and γH2AX in non-metastatic SCC (NMSCC) and metastatic SCC (MSCC). γH2AX in MSCC is lower magnification of Suppl. Fig. S2a. (b) Double immunofluorescence for keratin 10 (K10, red) and CE (green) in NMSCC. Right panel, amplified white square area. (c) Double immunofluorescence staining for p21 (red) and CE (green) in SCC. Note that both regulators are frequently co-expressed in individual cells (yellow), typical of a cell cycle conflict (21). Right panel with split colours amplifies white square area. (d) IHC staining for CE and p21 in serial sections of NMSCC. DAPI for DNA in blue. Scale Bars in a,b and d: 50µm. Scale bars in c: 50µm. It completes Fig. 2. Note that p21 is strong in large nuclei or multinucleate cells (arrows).

## Slide 6
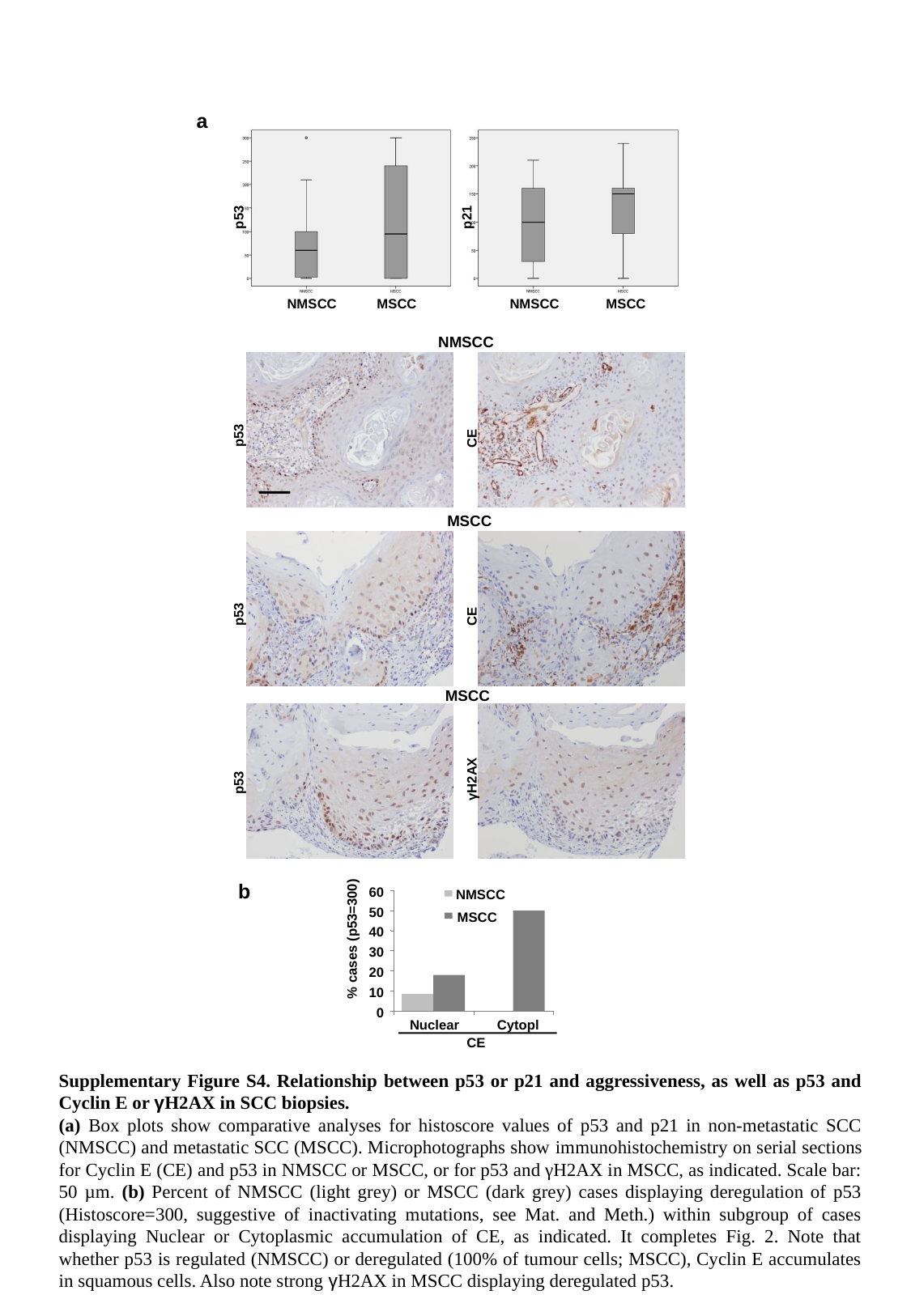

a
p53
p21
NMSCC
MSCC
NMSCC
MSCC
NMSCC
p53
CE
MSCC
p53
CE
MSCC
p53
γH2AX
b
60
NMSCC
50
MSCC
40
% cases (p53=300)
30
20
10
0
Nuclear
Cytopl
CE
Supplementary Figure S4. Relationship between p53 or p21 and aggressiveness, as well as p53 and Cyclin E or γH2AX in SCC biopsies.
(a) Box plots show comparative analyses for histoscore values of p53 and p21 in non-metastatic SCC (NMSCC) and metastatic SCC (MSCC). Microphotographs show immunohistochemistry on serial sections for Cyclin E (CE) and p53 in NMSCC or MSCC, or for p53 and γH2AX in MSCC, as indicated. Scale bar: 50 µm. (b) Percent of NMSCC (light grey) or MSCC (dark grey) cases displaying deregulation of p53 (Histoscore=300, suggestive of inactivating mutations, see Mat. and Meth.) within subgroup of cases displaying Nuclear or Cytoplasmic accumulation of CE, as indicated. It completes Fig. 2. Note that whether p53 is regulated (NMSCC) or deregulated (100% of tumour cells; MSCC), Cyclin E accumulates in squamous cells. Also note strong γH2AX in MSCC displaying deregulated p53.

## Slide 7
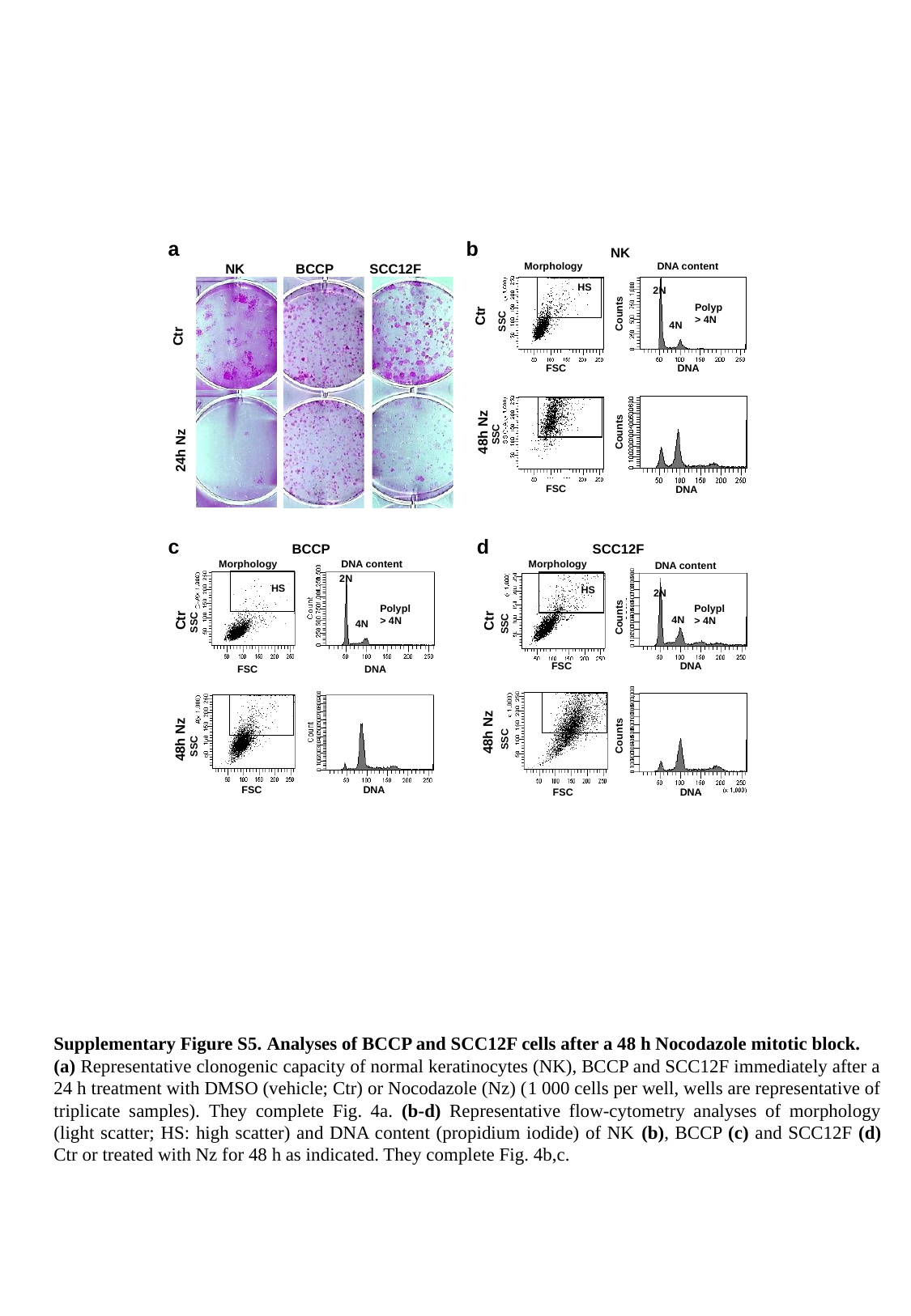

a
b
NK
Morphology
DNA content
NK
BCCP
SCC12F
Ctr
24h Nz
HS
2N
Polyp
> 4N
Ctr
Counts
SSC
4N
FSC
DNA
48h Nz
Counts
SSC
FSC
DNA
c
d
BCCP
SCC12F
Morphology
DNA content
Morphology
DNA content
2N
HS
HS
2N
Polypl
> 4N
Polypl
> 4N
Ctr
Ctr
SSC
SSC
Counts
4N
V
FSC
4N
FSC
DNA
DNA
48h Nz
48h Nz
SSC
Counts
SSC
V
FSC
FSC
DNA
DNA
Supplementary Figure S5. Analyses of BCCP and SCC12F cells after a 48 h Nocodazole mitotic block.
(a) Representative clonogenic capacity of normal keratinocytes (NK), BCCP and SCC12F immediately after a 24 h treatment with DMSO (vehicle; Ctr) or Nocodazole (Nz) (1 000 cells per well, wells are representative of triplicate samples). They complete Fig. 4a. (b-d) Representative flow-cytometry analyses of morphology (light scatter; HS: high scatter) and DNA content (propidium iodide) of NK (b), BCCP (c) and SCC12F (d) Ctr or treated with Nz for 48 h as indicated. They complete Fig. 4b,c.

## Slide 8
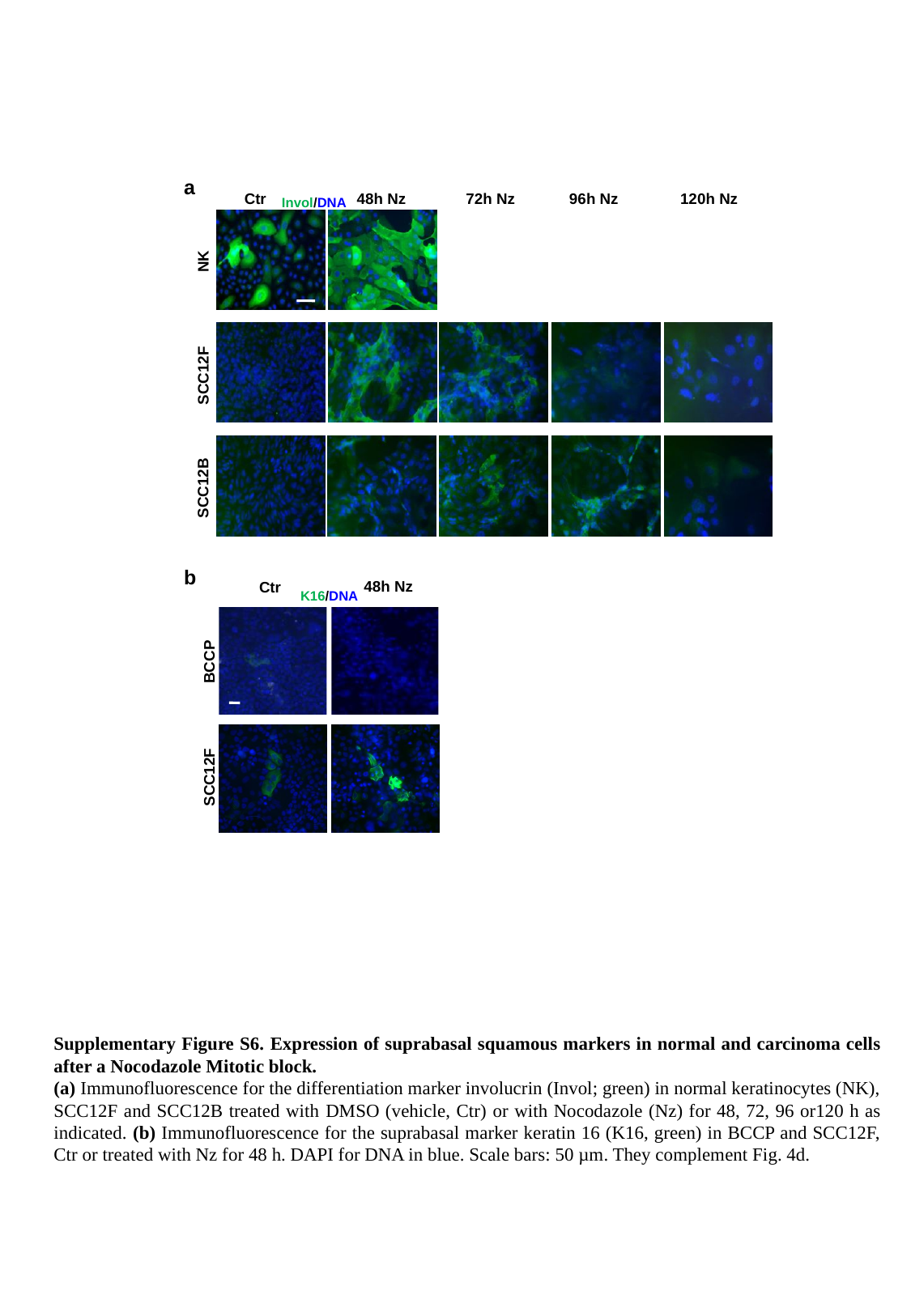

a
Ctr
48h Nz
72h Nz
96h Nz
120h Nz
Invol/DNA
NK
SCC12F
SCC12B
b
48h Nz
Ctr
K16/DNA
BCCP
SCC12F
Supplementary Figure S6. Expression of suprabasal squamous markers in normal and carcinoma cells after a Nocodazole Mitotic block.
(a) Immunofluorescence for the differentiation marker involucrin (Invol; green) in normal keratinocytes (NK), SCC12F and SCC12B treated with DMSO (vehicle, Ctr) or with Nocodazole (Nz) for 48, 72, 96 or120 h as indicated. (b) Immunofluorescence for the suprabasal marker keratin 16 (K16, green) in BCCP and SCC12F, Ctr or treated with Nz for 48 h. DAPI for DNA in blue. Scale bars: 50 µm. They complement Fig. 4d.

## Slide 9
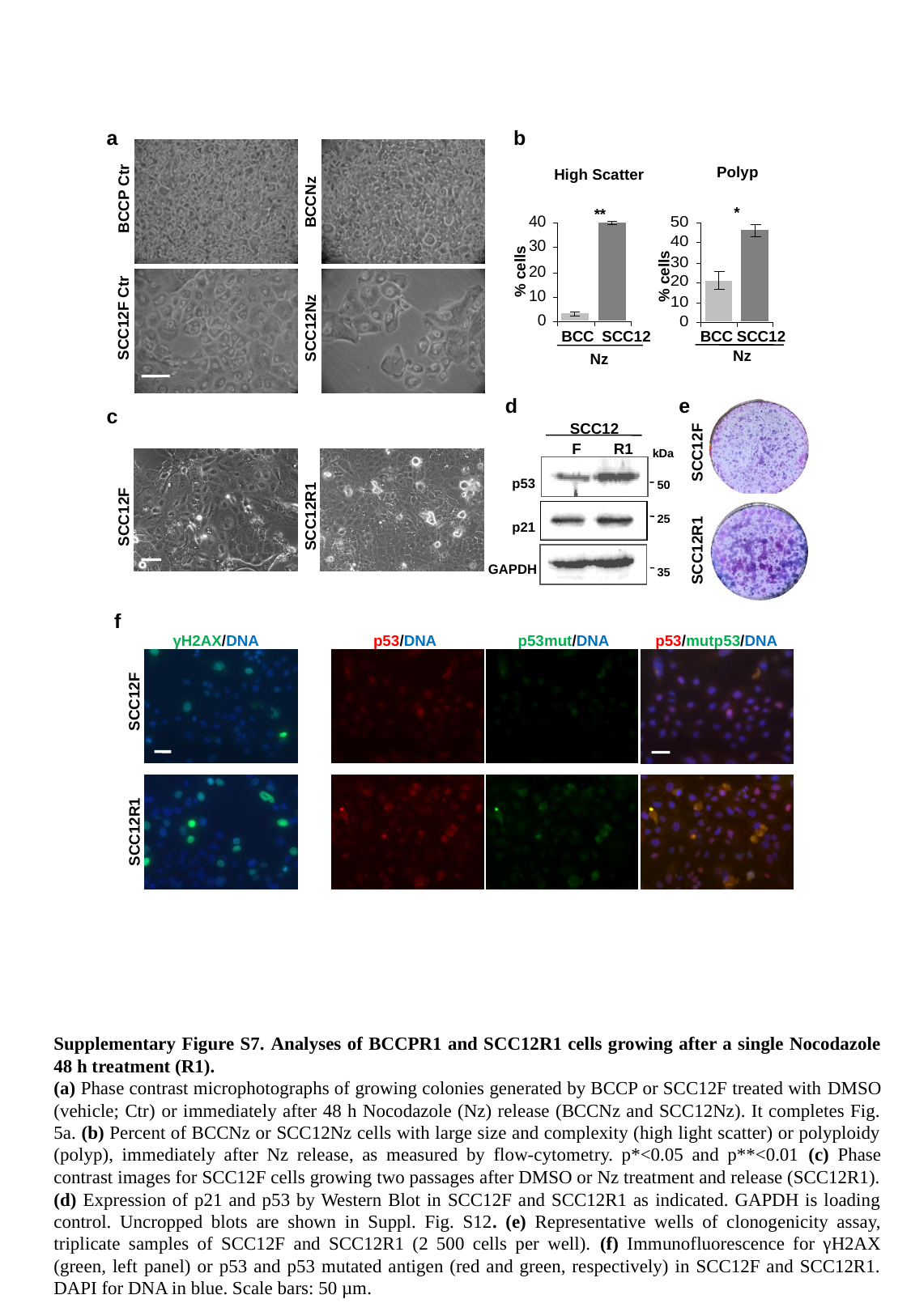

a
b
Polyp
*
% cells
BCC
SCC12
Nz
High Scatter
BCCNz
BCCP Ctr
**
% cells
SCC12F Ctr
SCC12Nz
BCC
SCC12
Nz
d
e
c
SCC12
SCC12F
F
R1
kDa
p53
50
SCC12R1
SCC12F
25
p21
SCC12R1
GAPDH
35
f
γH2AX/DNA
p53/DNA
p53mut/DNA
p53/mutp53/DNA
SCC12F
SCC12R1
Supplementary Figure S7. Analyses of BCCPR1 and SCC12R1 cells growing after a single Nocodazole 48 h treatment (R1).
(a) Phase contrast microphotographs of growing colonies generated by BCCP or SCC12F treated with DMSO (vehicle; Ctr) or immediately after 48 h Nocodazole (Nz) release (BCCNz and SCC12Nz). It completes Fig. 5a. (b) Percent of BCCNz or SCC12Nz cells with large size and complexity (high light scatter) or polyploidy (polyp), immediately after Nz release, as measured by flow-cytometry. p*<0.05 and p**<0.01 (c) Phase contrast images for SCC12F cells growing two passages after DMSO or Nz treatment and release (SCC12R1). (d) Expression of p21 and p53 by Western Blot in SCC12F and SCC12R1 as indicated. GAPDH is loading control. Uncropped blots are shown in Suppl. Fig. S12. (e) Representative wells of clonogenicity assay, triplicate samples of SCC12F and SCC12R1 (2 500 cells per well). (f) Immunofluorescence for γH2AX (green, left panel) or p53 and p53 mutated antigen (red and green, respectively) in SCC12F and SCC12R1. DAPI for DNA in blue. Scale bars: 50 µm.

## Slide 10
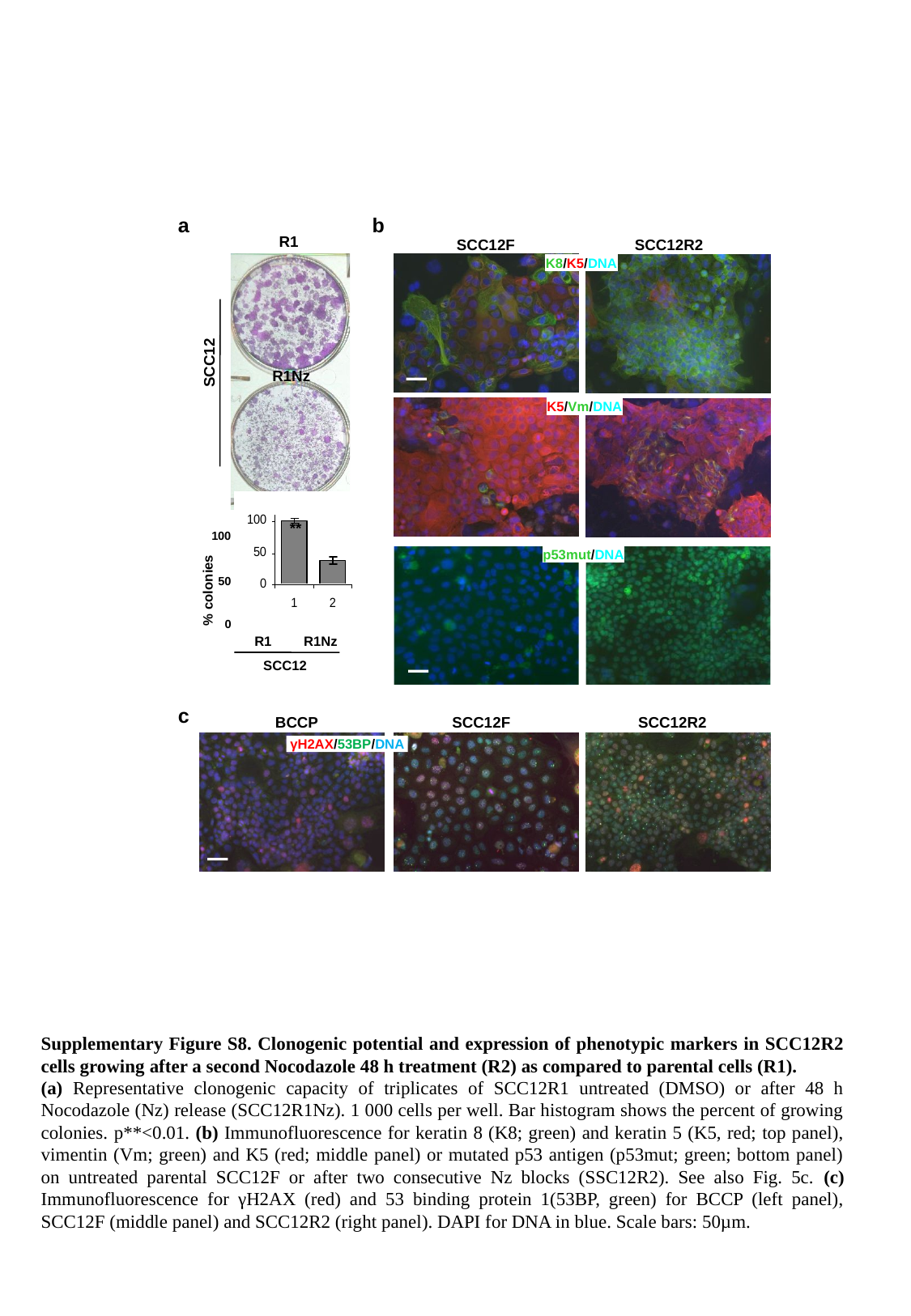

a
b
R1
SCC12F
SCC12R2
K8/K5/DNA
 SCC12
R1Nz
K5/Vm/DNA
**
100
p53mut/DNA
50
% colonies
0
R1
R1Nz
 SCC12
c
BCCP
SCC12F
SCC12R2
 γH2AX/53BP/DNA
Supplementary Figure S8. Clonogenic potential and expression of phenotypic markers in SCC12R2 cells growing after a second Nocodazole 48 h treatment (R2) as compared to parental cells (R1).
(a) Representative clonogenic capacity of triplicates of SCC12R1 untreated (DMSO) or after 48 h Nocodazole (Nz) release (SCC12R1Nz). 1 000 cells per well. Bar histogram shows the percent of growing colonies. p**<0.01. (b) Immunofluorescence for keratin 8 (K8; green) and keratin 5 (K5, red; top panel), vimentin (Vm; green) and K5 (red; middle panel) or mutated p53 antigen (p53mut; green; bottom panel) on untreated parental SCC12F or after two consecutive Nz blocks (SSC12R2). See also Fig. 5c. (c) Immunofluorescence for γH2AX (red) and 53 binding protein 1(53BP, green) for BCCP (left panel), SCC12F (middle panel) and SCC12R2 (right panel). DAPI for DNA in blue. Scale bars: 50µm.

## Slide 11
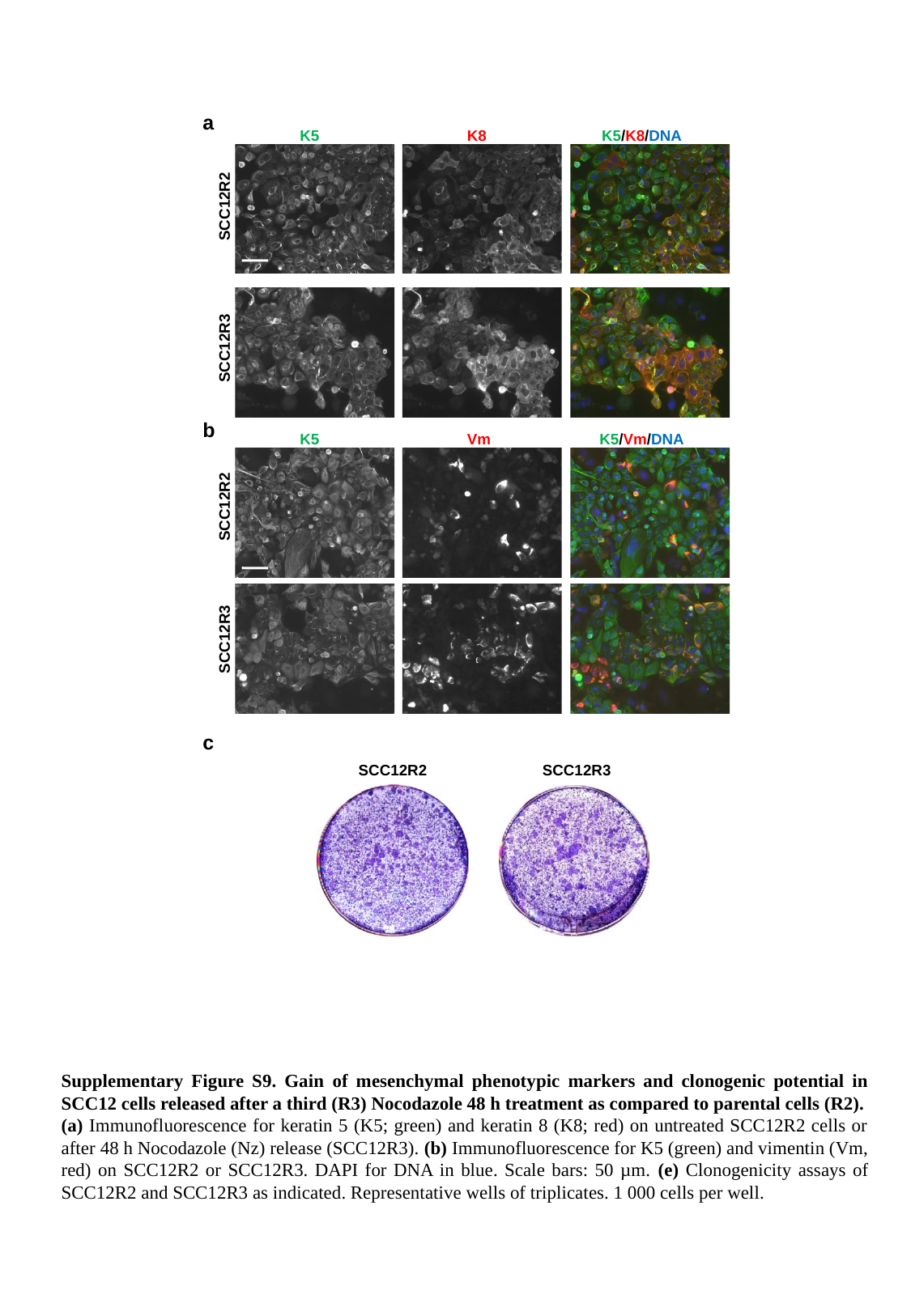

a
K5
K8
K5/K8/DNA
SCC12R2
SCC12R3
b
K5
Vm
K5/Vm/DNA
SCC12R2
SCC12R3
c
SCC12R2
SCC12R3
Supplementary Figure S9. Gain of mesenchymal phenotypic markers and clonogenic potential in SCC12 cells released after a third (R3) Nocodazole 48 h treatment as compared to parental cells (R2).
(a) Immunofluorescence for keratin 5 (K5; green) and keratin 8 (K8; red) on untreated SCC12R2 cells or after 48 h Nocodazole (Nz) release (SCC12R3). (b) Immunofluorescence for K5 (green) and vimentin (Vm, red) on SCC12R2 or SCC12R3. DAPI for DNA in blue. Scale bars: 50 µm. (e) Clonogenicity assays of SCC12R2 and SCC12R3 as indicated. Representative wells of triplicates. 1 000 cells per well.

## Slide 12
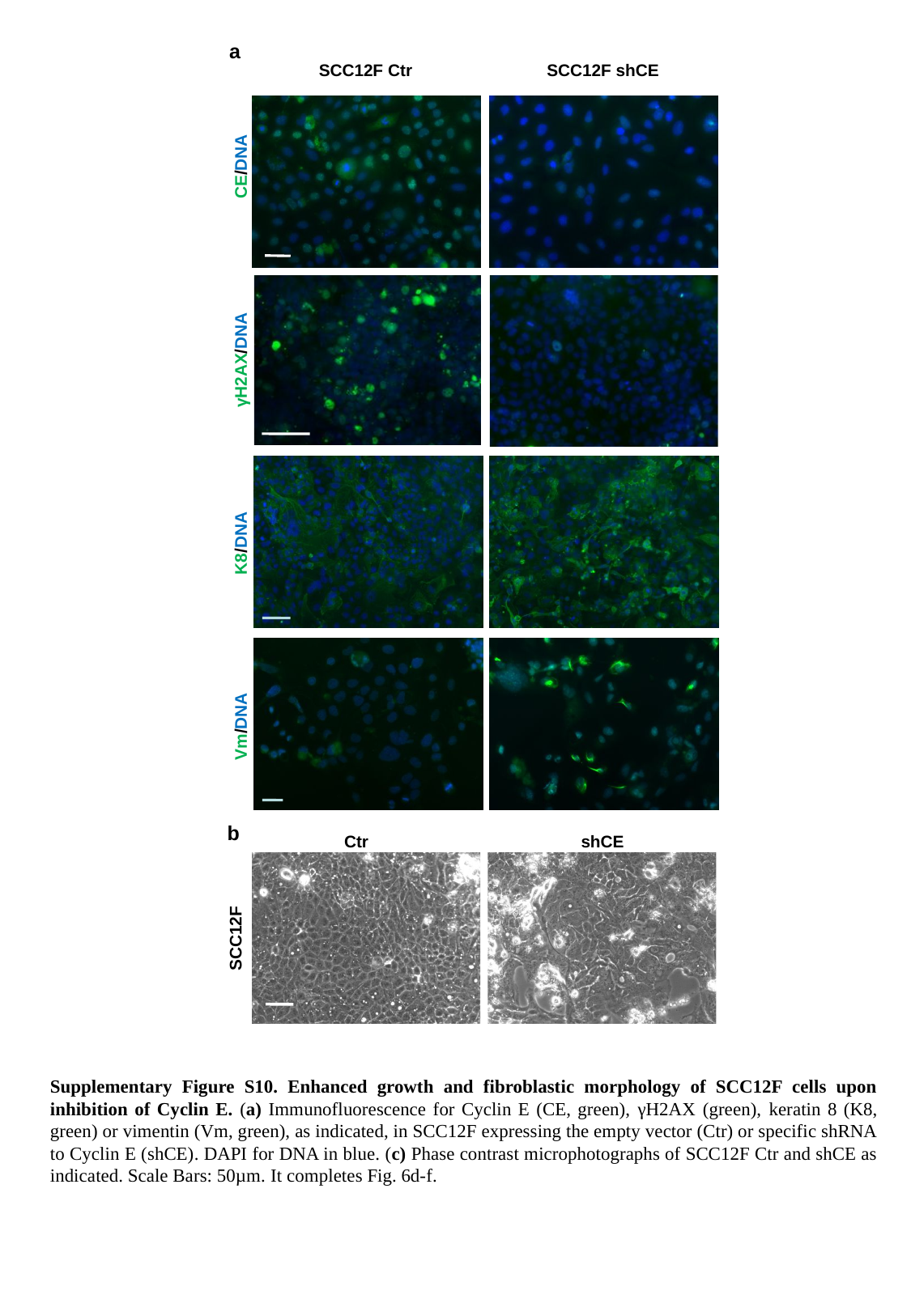

a
SCC12F Ctr
SCC12F shCE
CE/DNA
γH2AX/DNA
K8/DNA
Vm/DNA
b
Ctr
shCE
SCC12F
Supplementary Figure S10. Enhanced growth and fibroblastic morphology of SCC12F cells upon inhibition of Cyclin E. (a) Immunofluorescence for Cyclin E (CE, green), γH2AX (green), keratin 8 (K8, green) or vimentin (Vm, green), as indicated, in SCC12F expressing the empty vector (Ctr) or specific shRNA to Cyclin E (shCE). DAPI for DNA in blue. (c) Phase contrast microphotographs of SCC12F Ctr and shCE as indicated. Scale Bars: 50µm. It completes Fig. 6d-f.

## Slide 13
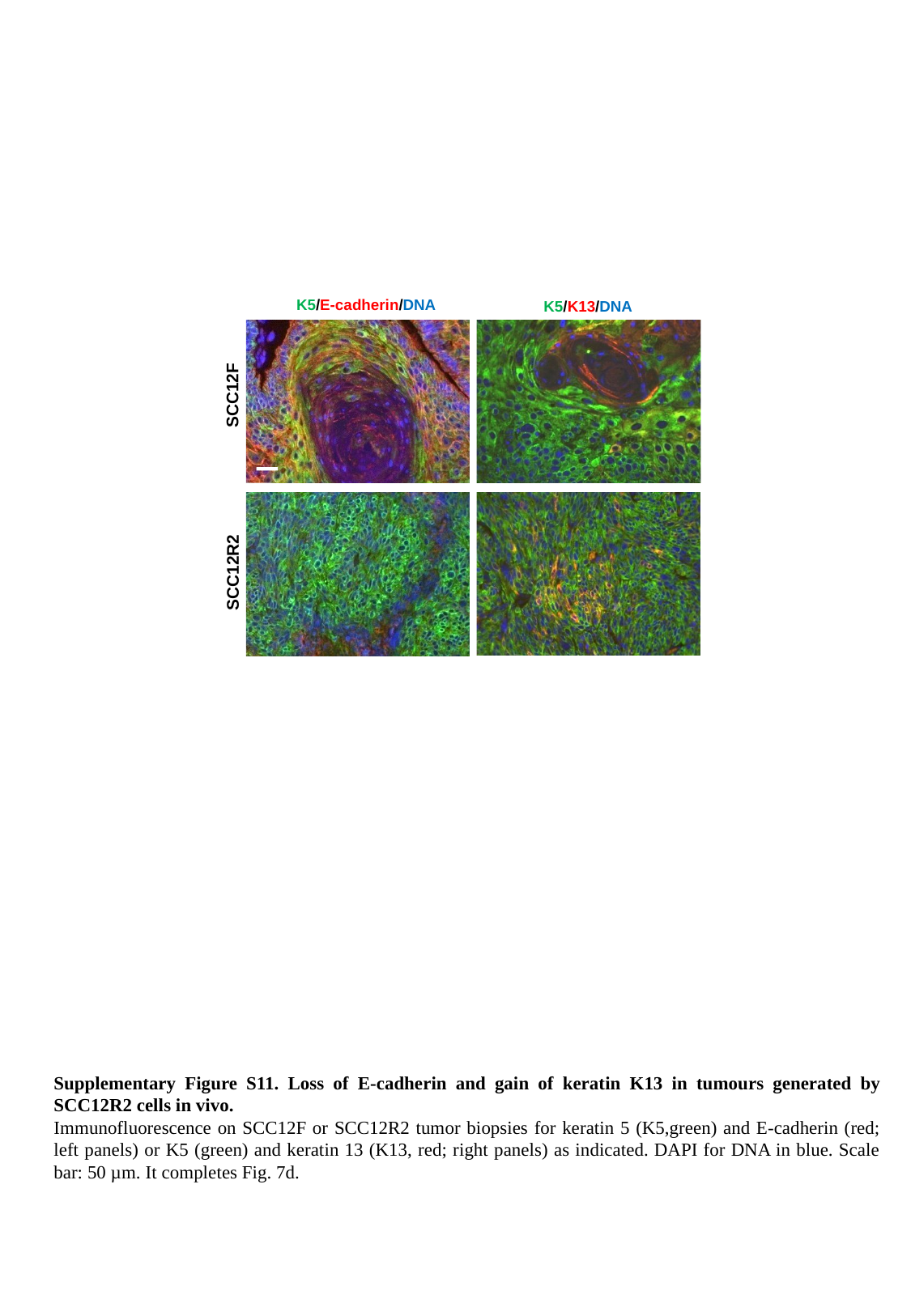

K5/E-cadherin/DNA
K5/K13/DNA
SCC12F
SCC12R2
Supplementary Figure S11. Loss of E-cadherin and gain of keratin K13 in tumours generated by SCC12R2 cells in vivo.
Immunofluorescence on SCC12F or SCC12R2 tumor biopsies for keratin 5 (K5,green) and E-cadherin (red; left panels) or K5 (green) and keratin 13 (K13, red; right panels) as indicated. DAPI for DNA in blue. Scale bar: 50 µm. It completes Fig. 7d.

## Slide 14
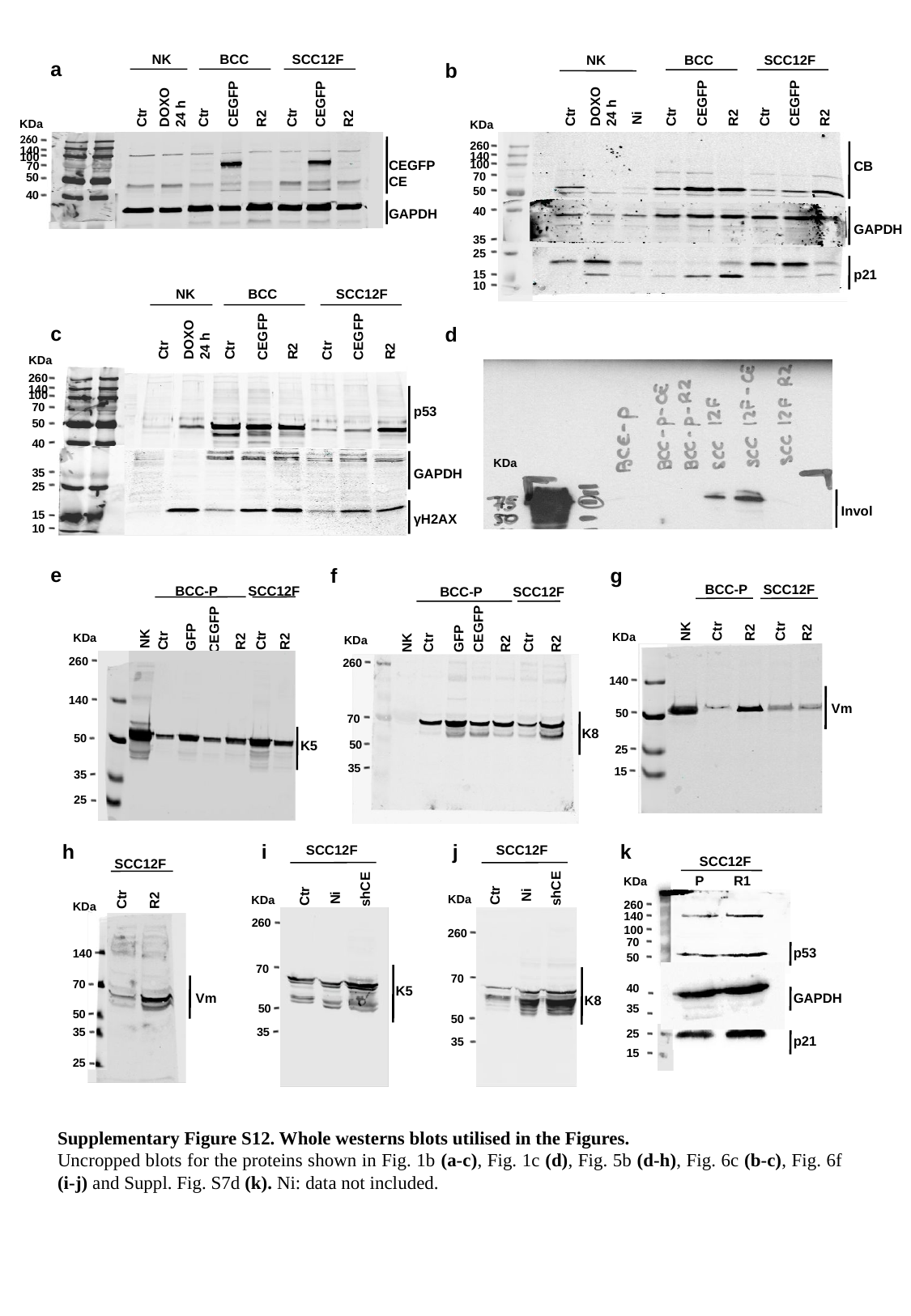

NK
BCC
SCC12F
CEGFP
DOXO 24 h
CEGFP
Ctr
R2
Ctr
R2
Ctr
KDa
260
CB
GAPDH
p21
140
100
70
50
40
35
25
15
10
NK
BCC
SCC12F
DOXO 24 h
CEGFP
CEGFP
Ctr
R2
Ctr
R2
Ctr
CEGFP
CE
GAPDH
KDa
260
140
100
70
40
a
b
Ni
50
NK
BCC
SCC12F
DOXO 24 h
CEGFP
CEGFP
Ctr
R2
R2
Ctr
Ctr
KDa
260
140
100
70
50
40
35
25
15
10
c
d
Invol
KDa
p53
GAPDH
γH2AX
e
f
g
BCC-P
SCC12F
BCC-P
SCC12F
BCC-P
SCC12F
NK
Ctr
R2
Ctr
R2
NK
CEGFP
NK
Ctr
GFP
R2
Ctr
R2
CEGFP
GFP
KDa
KDa
Ctr
Ctr
KDa
R2
R2
260
260
140
140
Vm
50
70
K8
50
50
K5
25
35
15
35
25
h
i
j
k
SCC12F
SCC12F
SCC12F
Ctr
R2
140
70
Vm
50
35
25
KDa
SCC12F
Ni
Ni
P
R1
KDa
shCE
shCE
Ctr
Ctr
KDa
KDa
260
140
260
100
260
70
p53
50
70
70
40
K5
GAPDH
K8
50
35
50
35
25
p21
35
15
Supplementary Figure S12. Whole westerns blots utilised in the Figures.
Uncropped blots for the proteins shown in Fig. 1b (a-c), Fig. 1c (d), Fig. 5b (d-h), Fig. 6c (b-c), Fig. 6f (i-j) and Suppl. Fig. S7d (k). Ni: data not included.
