# Supplementary material for: Inefficient differentiation response to cell cycle stress leads to genomic instability and malignant progression of squamous carcinoma cells
Source: Cell Death Dis. 2017 Jun 29;8(6):e2901–. doi: 10.1038/cddis.2017.259 (PMC5520915; doi:10.1038/cddis.2017.259)
Supplement: Supplementary Material and Methods [file cddis2017259x2.pdf]

SUPPLEMENTARY MATERIAL AND METHODS

Sample collection and characterization

Samples were retrieved from the departments of pathology of Hospital del Mar (Barcelona, Spain) and Clínica Mompía (Santander, Spain) and analysed each sample and registered the following histopathological features using hematoxylin and eosin stained slides: tumour differentiation (well, moderately, poorly), tumour thickness, desmoplasia (1), vascular and perineural invasion. Approval to conduct this study was obtained from the ethics committees from all the centres participating in the study, and in accordance with the guidelines of the Helsinki Declaration of 1975, as revised in 1983.

Lentiviral production

Transient transfection of producer 293T cells using jetPEI™ was made as the manufacturer indicated (PolyPlus-Transfection, Illkirch, France). 24 h after transfection, the culture medium was replaced by Rheinwald FAD medium and viral supernatant was made during around 8h. The supernatant was collected, filtered through a 0.22 µm mesh and completed with 8µg/ml Polybrene (Sigma-Aldrich). The viral supernatant was then immediately added to primary keratinocytes that had been plated in Rheinwald FAD medium (6 h before) and medium supernatant was replaced by fresh medium 12 h later.

Clonogenicity assays

For clonogenicity assays, 1 000 or 2 500 total cells were plated per T6 well triplicates and cultured in FAD medium. 7-10 days later, the cultures were stained with rhodanile blue as described previously (2). This dye colors keratinocytes pink and feeder fibroblasts purple.

Antibodies

The following primary antibodies from Santa Cruz Biotechnology were used: anti-Cyclin B (GNS1, lot J0108; Immunofluorescence, IF, Western blotting, WB) anti-Cyclin E1 (C19, lot C1914; IF), anti-Cyclin E1 (HE12, lot H2511; IF, WB and Immunohistochemistry, IHC), anti-GAPDH (FL-335 lot E0212; WB), anti-K10 (RKSE60, lot J5012; IF), anti-K16 (sc-53255, lot F2812; IF), anti-p53 (FL-393, lot A271; IF and WB) and anti-p53mutated (Pab24,

lot K1512; IF).The primary antibodies used from Sigma-Aldrich were: anti-involucrin (SY5, lot 071M4784; IF and WB), anti-K5 (lot 310246; IF and WB), anti-K8 (M20, C5301; IF and WB), anti-p21CIP (CP74; IF, WB and IHC) and anti-CD8 (lot C7423; Flow Cytometry, FC). Other antibodies used were: anti-53BP1 (A300-272A, Bethyl), anti-BrdU (347580, lot 36576; BD Biosciencies; FC), anti-E-Cadherin (BD Biosciences, IF), anti-GFP (lot 1141866; Invitrogen; IF and WB), anti-phospho-Histone H2AX (Ser139, JBW301 lot 1997719; Millipore; IF, WB, and IHC), anti-K1 (lot 09KCO2022; Covance; IF), anti-K5 (Covance; IF), anti-K8 (Troma; IF), anti-K13 (Abcam; IF), anti-Ki67 (Thermo Scientific, IHC) and anti-vimentin (NCL-VIM-V9, lot 6015148; Leica-Novocastra; IF and WB). The following secondary antibodies from Jackson ImmunoResearch were used: Alexa Fluor® 488-conjugated goat anti rabbit or anti mouse IgG antibodies (FC and IF); Alexa Fluor® 594-conjugated goat anti rabbit or anti mouse IgG antibodies (IF); and biotin-coupled goat anti rabbit or anti mouse IgG antibodies (IHC). Other secondary antibodies used were IRdye800-conjugated goat anti rabbit or anti mouse IgG antibodies (Li-Cor, WB) and HRP-conjugated goat anti rabbit or anti mouse IgG antibodies (Bio-Rad, WB).

Flow Cytometry

Cells were harvested, fixed and stained as previously described for DNA synthesis and content (BrdU incorporation and propidium iodide). For DNA synthesis analyses, cells that had been cultured in the presence of 10μM BrdU (Sigma-Aldrich) for 1.5 hours, were harvested, fixed and stained for BrdU and DNA with propidium iodide (PI; 25 μg/ml, 12h) as described (3). After staining, cells were firmly resuspended and filtered through a 70 μM mesh to minimize the presence of aggregates and then analyzed on a Becton Dickinson BD FACScan™. A total of 10 000 events were acquired and in order to gate out aggregates, the area of the fluorescent pulse of PI (DNA content; PE-A) was plotted versus the width of the fluorescent pulse (PE-W).

Primers used for RT-PCR analyses

Human Keratin 5 (K5)

5′-CAAGCGTACCACTGCTGAGA-3′

5′-CTATCCAGGTCCAGGTTGCG-3′

Human Vimentin (Vm)

5′-GGACCAGCTAACCAACGACA-3′

5′-AAGGTCAAGACGTGCCAGAG-3′

Human β-Actin (Act)

5′-AAAATCTGGCACCACACCTTC-3′

5′-AGCACAGCCTGGATAGCAA-3′

Mice conditions

Six-week-old female BALB-nu/nu mice were purchased from Charles River France, Inc. (Saint-Germain-sur-l'Arbresle, France). Mice were housed in a pathogen-free environment under controlled conditions (temperature 20–26°C, humidity 40–70%, light-dark cycle 12–12 h). Chlorinated water and irradiated food were provided ad libitum. The animals were allowed to acclimatize and recover from shipping-related stress for one week prior to the study.

Histology and Immunostaining

Sections were deparaffinized using standard protocols, and heat-induced antigen retrieval was performed in 0,01 M sodium citrate (pH 6) for 15 min in a pressure cooker or in microwave boiling in citrate buffer. The slides were then incubated with primary antibodies for 12 h at 4ºC. Immunohistochemistry sections were incubated with appropriate biotin-coupeled secondary antibodies, followed by avidin-peroxidase (ABC elite kit Vector, Burlingame, CA, USA). Positive staining was determined using diaminobenzidine as a substarte (DAB kit Vector, Burlingame, CA, USA). Immunofluorescence sections were incubated with the appropriate secondary antibodies. After washing with PBS, coverslips and slides were stained with 0.1μg/ml DAPI, mounted with Prolong Gold Antifade Reagent (Life Technologies), and visualized and photographed under AxioVision Zeiss fluorescent microscopy.

References

1. Brantsch KD, Meisner C, Schonfisch B, Trilling B, Wehner-Caroli J, Rocken M, et al. Analysis of risk factors determining prognosis of cutaneous squamous-cell carcinoma: a prospective study. Lancet Oncol. 2008;9(8):713-20. Epub 2008/07/12.
2. Jones PH, Watt FM. Separation of human epidermal stem cells from transit amplifying cells on the basis of differences in integrin function and expression. Cell. 1993;73(4):713-24.
3. Freije A, Ceballos L, Coisy M, Barnes L, Rosa M, De Diego E, et al. Cyclin E drives human keratinocyte growth into differentiation. Oncogene. 2012;31(50):5180-92.
